# Dynamics of the impact of COVID-19 on the economic activity of Peru

**DOI:** 10.1371/journal.pone.0244920

**Published:** 2021-01-08

**Authors:** Luis Varona, Jorge R. Gonzales

**Affiliations:** 1 Comillas Pontifical University of Madrid, Madrid, Spain; 2 National Autonomous University of Mexico, Castillo, Peru; The Bucharest University of Economic Studies, ROMANIA

## Abstract

**Background:**

The COVID-19 virus impacts human health and the world economy, causing in Peru, more than 800 thousand infected and a strong recession expressed in a drop of -12% in its economic growth rate for 2020. In this context, the objective of the study is to analyze the dynamics of the short-term behavior of economic activity, as well as to explain the causal relationships in a Pandemic context based on the basic number of spread (R_e_) of COVID-19 per day.

**Methods:**

An Autoregressive Distributed Lags (ARDL) model was used.

**Results:**

A negative and statistically significant impact of the COVID-19 shock was found on the level of economic activity and a long-term Cointegration relationship with an error correction model (CEM), with the expected sign and statistically significant at 1%.

**Conclusion:**

The Pandemic has behaved as a systemic shock of supply and aggregate demand at the macroeconomic level, which together have an impact on the recession or level of economic activity. The authors propose changing public health policy from an indiscriminate suppression strategy to a targeted, effective and intelligent mitigation strategy that minimizes the risk of human life costs and socioeconomic costs, in a context of uncertainty about the end of the Pandemic and complemented by economic, fiscal and monetary policies that mitigate the economic recession, considering the underlying structural characteristics of the Peruvian economy.

## 1. Introduction

COVID-19 has become an epidemiological and economic Global Pandemic [1]. For developing countries such as Peru, the impact is twofold: an external shock and an internal shock that affect aggregate supply and demand. The external shock implies a contraction in the prices of raw materials, in the demand for exports, employment, income, tourism, international remittances, and external financing.

The internal shock, being of greater impact, is associated with the COVID-19 disease due to the suppression policy to prevent contagion (quarantine), which affects employment and aggregate supply and, therefore, affects aggregate demand through lower consumption and savings or debt and private credit in the short-term; and lower investment and capital accumulation in the long-term, which is evident in the dynamics of economic activity [2].

From Barro, it is possible to say that a productivity shock generates co-movements in macroeconomic aggregates [3]. The key feature is that an epidemic naturally generates negative changes in both consumer demand and labor supply. These changes appear because consumption and work increase the risks of infection for people who are not immune to the virus [4–6].

The International Monetary Fund (IMF) projects negative growth for all regions in 2020 [7]. However, there are important differences between countries. There is a scenario of generalized recession at the level of developed countries and in developing countries, it is greater. The main economies have fallen in the first quarter of the year on average -5%, and the strongest economies such as the United States (US) and European Union (UE) countries are expected to fall to -7.1% and -9.3% respectively for the year 2020. Peru could fall more than -14% by 2020 [7]. This scenario adjusts to a global drop in economic activity of -4.4% for the second quarter of 2020 [8].

COVID-19 is a contagious disease that has generated high economic and social costs in the world [9]. In Peru, even with the suppression policy adopted, its impact is negative on both public health and economic activity [2, 10]. The objective of this research is to analyze the dynamics of the behavior of economic activity and COVID-19, as well as to explain the causal relationships and suggest public policy prescriptions for the resilience and reactivation of economic activity in Peru.

The methodology used here is the ARDL econometric model [11, 12], based on a macroeconomic model that considers COVID-19 as a systemic shock, which affects aggregate supply and aggregate demand in the New Keynesian style [13–15]. The paper is organized as follows: the first section includes a short review of the literature, the macroeconomic model, the stylized facts, and the Covid-19 in Latin America; the second section presents the methodology that includes an econometric model; the third section presents the main results; the fourth section discusses the main results and finally, the conclusions.

### Literature review

According to the review of the scientific economic literature, there is academic production that explains the level of economic activity and economic growth in the long-term [16–25]. However, there is little literature and empirical evidence for the short term, associated with Pandemic-type catastrophes such as COVID-19 with its daily dynamics. However, there are studies where epidemiological [26], climatic factors: rainfall [27, 28], temperature [29–31] and environmental [32, 33] are recognized, which emphasize the impact of economic activity. According to the SIR model, it has proven to be a good indicator of the evolution of COVID-19 [34].

Recent works try to explain the impact of COVID-19 on public health and economic activity, and also prescribe the public policies that could be implemented. Pioneering work [9]; the double curve [1]; the optimal trade-off between saving lives or the economy [5, 35–37] y Spence [38] with his study *Graphing the pandemic economy* that motivated this research. In the Peruvian case [34], there is a need to change strategy and solve structural problems in education and health. The Central Reserve Bank of Peru (BCRP) as a unique case of intervention, measures have been taken to deal with the pandemic [2, 34].

A team of epidemiologists from Imperial College shows that the global impact of COVID-19 has been profound and threatens public health, considered the most serious since the 1918 influenza pandemic. The team assesses the potential role of public health measures aimed at reducing contact rates in the population and thereby reducing virus transmission. They apply a simulation model in the UK (Great Britain) and the US. They conclude that the effectiveness of any intervention requires combining multiple interventions to have a substantial impact on transmission. With this, two strategies are possible: *mitigation and suppression* [9].

For Balwin, this economic crisis of COVID-19 is different, affecting all the G7 nations and China. He argues that governments should focus on using fast and expensive measures to ensure that the circular flow of money is not interrupted. He maintains that the objective should be to reduce the crisis and prevent the economic fabric from being affected. The recession of the economy is a necessary public health measure. Keeping workers away from work and consumers away from consumption reduces economic activity and thus inevitably flattens the infection curve but tends to increase the macroeconomic recession curve [1].

The author suggests that COVID-19 would cause three types of economic shocks: purely health shocks, the economic impacts of containment measures, and expectations shocks. He argues that countries are struggling to develop measures to flatten the recession curve and minimize the damage that this shock causes to the economy. It prescribes that economic policies to face the recession in economic activity can be grouped into fiscal policies, monetary policies, financial regulation policies, social security policies, industry policies, and trade policies.

Eichenbaum, Rebelo, and Trabandt extend the epidemiology model to study the interaction between economic decisions and epidemics. The model implies that people's decision to reduce consumption and work reduces the severity of the epidemic, measured by total deaths. They argue that the competitive equilibrium is not socially optimal because infected people do not fully internalize the effect of their economic decisions on the spread of the virus. According to the reference model, the best containment policy increases the severity of the recession but would save roughly half-a-million lives in the US [5].

The authors consider that there is a trade-off between the severity of the short-term recession caused by the epidemic and the consequences for health. They argue that the model considers forces that could affect the long-term performance of the economy such as bankruptcy costs, hysteresis effects of unemployment, and the destruction of supply chains. They recommend incorporating these forces into macroeconomic models of epidemics and studying their positive and normative implications. They conclude that the epidemic generates both supply and demand effects on economic activity and that these effects work together to generate a major recession.

Alvarez, Argente, and Lippi use the SIR epidemiological model. They argue that the optimal policy depends on the ratio of infected to susceptible in the population. Parameterize the model using data on the pandemic and the economic breadth of the lockdown. It assumes a severe blockage that begins two weeks after the outbreak, covers 60% of the population after one month, and gradually retreats, covering 20% of the population after 3 months. They conclude that welfare under said optimal policy is equivalent to a single payment of 2% of GDP [35].

Along these lines [36] developed a multigroup version of the epidemiological model based on the SIR model. It focuses on identifying the benefits that arise from optimal and specific policies that differentially block different groups. To do this, use three groups: young, middle-aged, and old. They argue that policymakers face trade-offs, such that when the priority is saving lives (*"security-centric" approach*), the economy will have to endure a prolonged lockdown and a significant decline in GDP. Conversely, if the economy is prioritized (*"economy-centric" approach*) and they try to keep economic damage to less than 10% of GDP for a year, they may be forced to endure a mortality rate of more than 1%. They conclude that measures that reduce interactions between groups, increasing testing, and isolating those infected can minimize both economic losses and deaths and serve as an application for future pandemics.

Loayza [37], argues that the pandemic crisis affects low- and middle-income countries disproportionately because most lack the resources and capacity to deal with a systemic shock. That developing countries, having more limited resources and capacities, but also younger populations, face different trade-offs in their fight against COVID-19 than advanced countries. In countries with higher populations and incomes, suppression measures may be optimal; whereas, for the poorest and youngest countries, more moderate measures may be better. It concludes that the goal of saving lives and livelihoods is possible with economic and public health policies adapted to the reality of developing countries. It points out that "smart" mitigation strategies (such as protecting the vulnerable and identifying and isolating the infected) pose substantial challenges for implementation, a combination of adaptation ingenuity is needed, a renewed effort by national authorities, and the support of the international community.

Specifically, for Peru, he affirmed that the suppression measures have been indiscriminate and that they have affected low-income populations, being a disaster not only economic and health but also humanitarian. And that the economic costs they suffer are associated with informality, a small tax base, an increase in the fiscal deficit, and governance. In addition, there is a lack of adequate incentives to motivate the population to comply with sanitary provisions, and that there are scarce basic health and housing conditions, as well as an increase in displacements in full quarantine motivated by need and culture, in the face of the demand to want work [39].

Spence [38], shows that an economic crisis evolves rapidly like a pandemic and that economic activity like other conventional economic variables is slow to be useful to policymakers on when to block and reopen sectors of the economy. The challenge for policymakers is to get the right balance to contain the virus and create the conditions for economic recovery.

In Peru [34], they study the behavior dynamics of COVID-19, based on the theoretical-mathematical model SIR, in addition to estimating and evaluating the impact of the suppression strategy as a public policy that has allowed to reduce the infected curve by more than 50%, but it has not been enough to mitigate the collapse of the Health System. This measure generates high social and economic costs due to the previous existence of structural characteristics, weak governance, State failures, and Market failures. To this is added corruption and institutional weakness, which generates higher costs. It concludes that the suppression strategy can be effective if it is implemented early and considers the structural characteristics of the economy. It recommends changing the suppression strategy that only leads to a deep recession in the economy.

The Government of Peru applies expansionary fiscal and monetary policies in the economy. The BCRP has implemented measures to safeguard the economy against the effects of COVID-19 [10]. The measures are aimed at reducing the cost of financing, providing liquidity to the financial system, and reducing the volatility of long-term interest rates and the exchange rate. It stimulates domestic demand to avoid the breakdown of the payment chain and gives relief to families and companies so that their ability to access credit is not affected [2]. And even economic reactivation Programs such as "*Reactiva Perú*" [40].

A more specialized review of the macroeconomic models related to the impacts of the Pandemic as a systemic shock. Models that consider that there are co-movements on the side of aggregate supply and aggregate demand [3], with assumptions of monopolistic competition and rigid prices [4, 13, 41], in addition to the assumption of capital accumulation [42]. And with limited access to rigorous information due to the characteristics of the Pandemic [43]. The authors show results found in the context of a rigid economy, an increase in the depth of the recession in the economy, and this applies to the Peruvian economy. Furthermore, the trend towards a fall in inflation can be seen [10] with a clear impact on income, wealth, consumption, and employment [44].

Barro recognizes that in the absence of aggregate productivity shocks, it is difficult for many models to generate co-movements in macroeconomic aggregates [3]. Gali [41] presents a basic New Keynesian model, with two key assumptions. Firstly, the market for goods is not perfectly competitive, therefore, monopolistic competition is assumed. Second, there is no full price flexibility that applies to small, open economies. He points out that it is difficult to think of a macro alternative to the economic paradigm that would eliminate the two defining characteristics of the New Keynesian model: nominal rigidities and not monetary neutralities [13].

The author acknowledges that the neoclassical model does not rationalize the positive co-movement of consumption and investment. He introduces monopolistic competition into the neoclassical model and remedies this deficiency even when prices are completely flexible. When prices are sticky they lead to a bigger recession but do not alter the predictions of the monopolistic competition model [4]. Therefore, he proposes an extension of the neoclassical model to include contagion dynamics, to study and quantify the tradeoffs of policies that can mitigate the COVID-19 pandemic. And this model reveals i) that, in relation to the incentives of private agents, a planner wishes to anticipate mitigation strategies; ii) the prospect of mitigation together with the possibility for agents to work from home significantly reduces the spread of the disease and the economic costs [42].

In the US, a survey with more than 10.000 respondents found that 50% of participants report loss of income and wealth due to COVID-19, with average losses of US$ 5.293 and US$ 33.482 respectively, and the aggregate expense of consumers fell by 31 percentage points. They also expect lower future inflation, reporting greater uncertainty. While the lockdowns have effects on local economic conditions and household expectations, they have little impact on Congress, FED, or Treasury approval ratings [44].

There are limitations to the production of rigorous statistical information. The current conditions of the pandemic have not made it possible to ensure the production of high-quality economic statistics on key variables in a rigorous way. It happens for the IMF, EUROSTAT, and the UN [43]. However, theoretical models that attempt to describe and explain the impacts of COVID-19 on economic variables have used statistics or operational variables obtained from the Web. And these methodologies have been used with Google Trend with variables of economic activity [45–47] and for some disease or pandemic [48–51].

From a brief review of the literature, we consider a macroeconomic model that has COVID-19 as a systemic shock that affects both aggregate demand and aggregate supply. It can be seen in the reality that prices tend to be rigid. For greater detail, the theoretical-mathematical model is derived as support information (S1 Model). Next, we use the graphical model to explain the impact of COVID-19 on the economic activity of Peru.

### COVID-19 and aggregate supply and demand shocks

**Fig 1:**
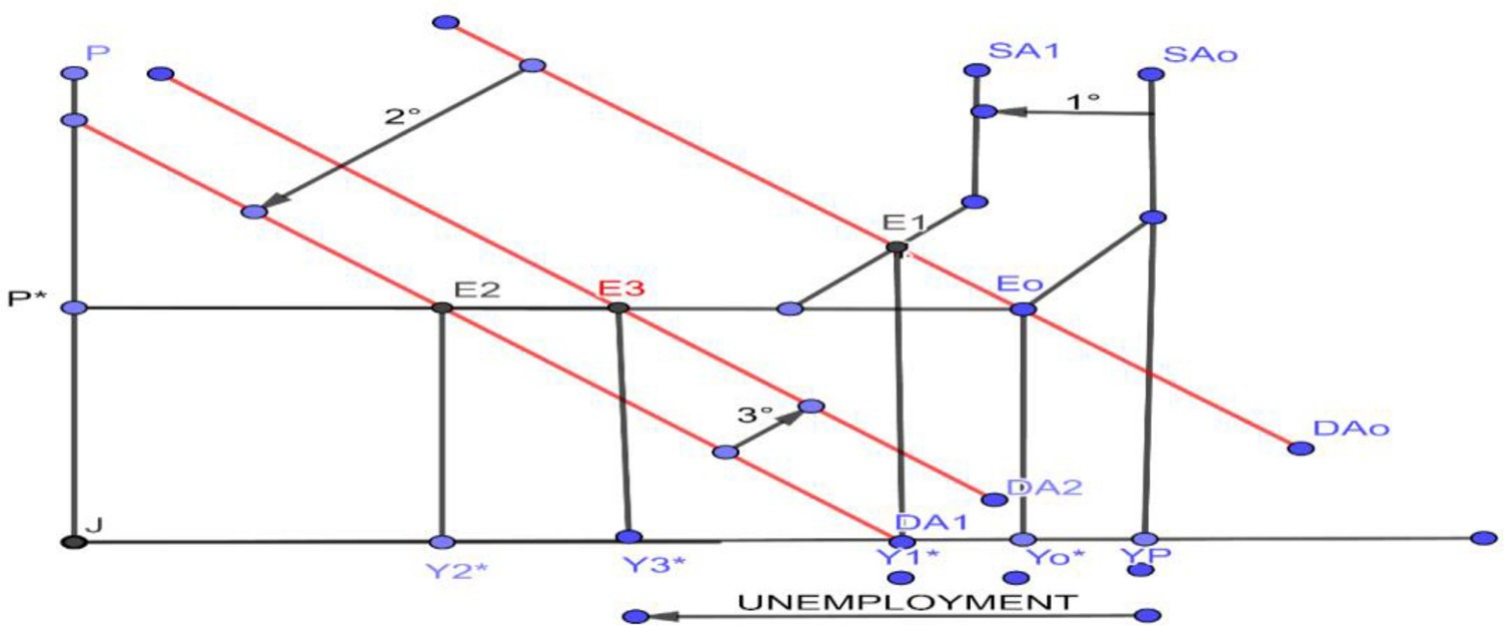
Short-term macroeconomic equilibrium. COVID-19 shock in aggregate supply (AS) and aggregate demand (AD). Source: Own elaboration, from Dornbusch et al. (2011); Blanchard, O. (2013), Galí (2015, 2018); Mankiw, Phelps, and Romer (1995); Stiglitz (2015) and Samuelson (1958). FP unemployment indicates the unemployment of production factors.

Economic science makes use of the theoretical-mathematical model, graphical model, and econometric model to describe, explain, and forecast the impact of exogenous variables or shocks such as a pandemic. COVID-19 has impacted aggregate supply and demand shock in economic activity. In Fig 1, the representation of macroeconomic equilibrium based on the equality of Aggregate Supply (SA) and Aggregate Demand (DA) is observed. A small, volatile economy is assumed, which depends on international and dual markets, with a labor market where the informal sector prevails, with large differences in productivity, wages, and income. Likewise, an economy is assumed that in the short-term has fixed prices in the Keynesian style [13, 14, 41]. Fig 1 shows the initial equilibrium of the economy that is achieved at point (E_0_), where aggregate supply (SA_0_) and aggregate demand (DA_0_) intersect, which determine the equilibrium prices (P*) and the level of economic activity (Y_0_*). Given the pandemic situation, which operationally is reflected in the increase of the variable, basic spread number (R_e_), leads policy-makers to implement a suppression strategy (quarantine), which implies that the workforce does not they work, they do not produce and their income is reduced, therefore, the aggregate supply is affected, moving from SA_0_ a SA_1_. This leads to a new E_1_ equilibrium, in which it is evident that macroeconomically, the level of employment, income levels, and increased unemployment of productive factors and informality in the economy have fallen.

The lower levels of income reduce the level of consumption and savings of families, likewise, complemented by external effect, the reduction of income in developed countries, generate a reduction in our exports of raw materials, the reduction of international tourists and therefore, contraction of tourist activities, etc., which further recession the economy, shifting aggregate demand from DA_0_ a DA_1_ and a new equilibrium is observed in E_2_, with a level of income and product of equilibrium Y_2_*, which reflect a greater economic recession.

Likewise, after the public health policy of suppression, it can be seen that the R_e_ parameter begins a decreasing trend but greater than unity, showing at the same time a deep economic recession and a persistent increase in the number of infected and deaths. Given this situation, the Government begins a process of implementing *Keynesian policies* to expand current spending in the short term to stimulate economic activity (money transfers, bonds, and the process of economic reactivation through the resumption of activities in four phases and the implementation of the *'Reactiva-Peru'* Program [40]) and likewise, the BCRP implements expansionary monetary policies (increase in money issuance, credit expansion, reduction of real interest rate, open market operations), which revert and impact on less measure of aggregate demand [2].

This implies that in a third moment the aggregate demand has been gradually shifting from DA_1_ to DA_2_, reaching an income level of equilibrium Y_3_*, income or GDP that still reflects a high level of recession and unemployment of productive factors in the economy a lot lower than levels before the start of the pandemic.

Likewise, it is observed that the R_e_ parameter is gradually reversed as the formal and informal labor force begin to resume their economic activities with the process of economic reactivation in four phases [52, 53]. Experts predict that the recovery of the economy could take one to two years (Y_0_*) and obtain levels of GDP similar to the levels before the pandemic. For Seminario, the fall of the Peruvian economy would be due to dependence on the metal export model [54] but rather due to the COVID-19 pandemic that closes the Neoliberal period [55]. Likewise, epidemiologists maintain that there is no end date for the pandemic, which will depend on the finding of the vaccine against COVID-19. However, the model describes the co-movements of both aggregate supply and aggregate demand for the Peruvian pandemic economy [3, 4], based on the new Keynesian theory [13, 15, 24, 25, 41, 56, 57].

### Stylized fact

**Fig 2:**
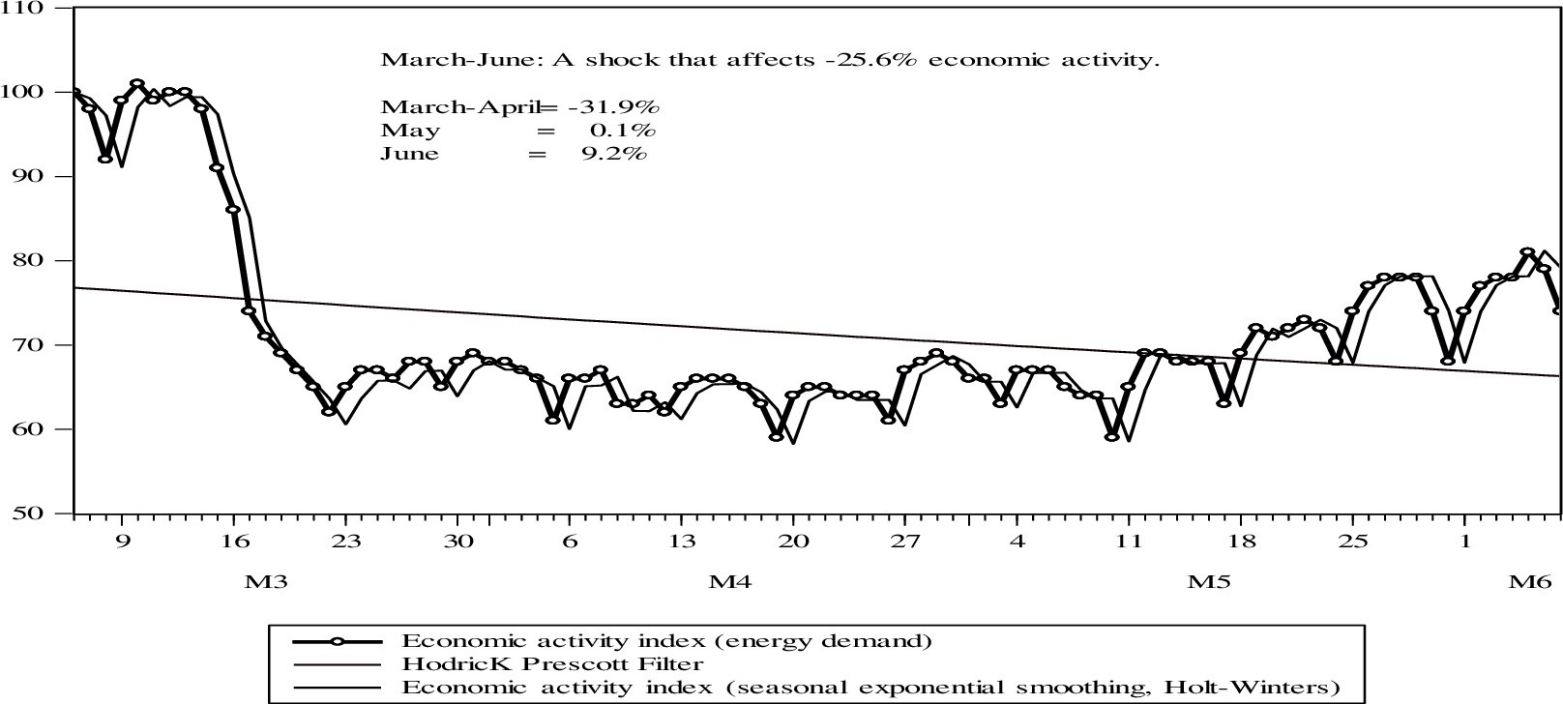
Economic activity index, based on energy demand (Ye) in average kilowatts/day (KW). In index with the base date March 6 = 100. Analysis period from the start of the pandemic March 6 to June 7, 2020. Source: COES. Own elaboration.

In Peru, there is evidence that economic activity has fallen drastically and has gradually recovered. In Fig 2 it can be seen that the COVID-19 pandemic has caused an adverse external shock, but also a negative internal shock between March-June that has resulted in a fall of more than -25% in economic activity. This is explained by variables of aggregate supply and aggregate demand, the COVID-19 shock, the suppression strategy implemented by the Government as of March 13, 2020. There are signs to believe that the impact of the pandemic on activity economic could be even higher. According to the National Institute of Statistics and Informatics (INEI), between March and July 2020, the monthly growth rates of GDP were -16.7%; -39.9%; -32.7%; -18.1%, and -11.7% respectively, showing that they have decreased gradually as of April.

**Fig 3:**
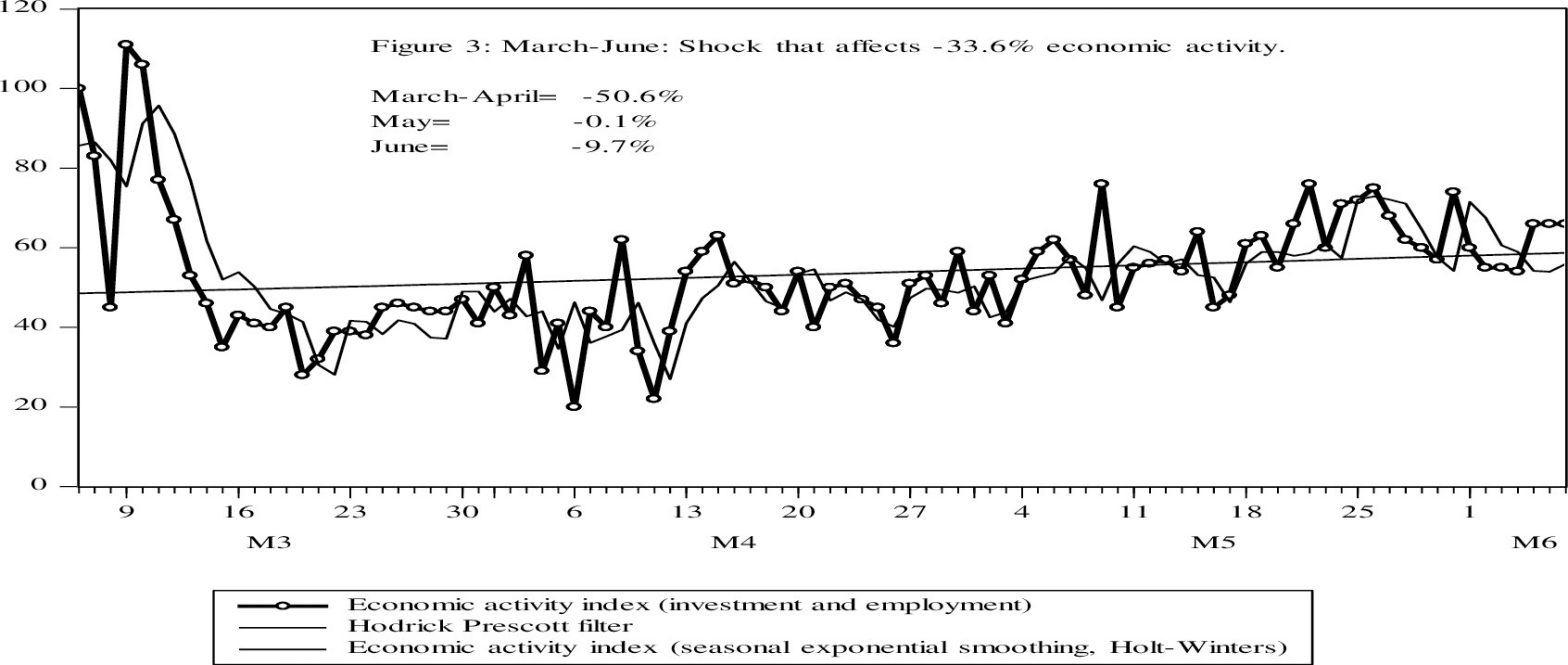
Economic activity index, from employment (Yw), and investment and employment search (Yi), for Google search. In index with the base date March 6 = 100. Analysis period from the start of the pandemic March 6 to June 7, 2020. Source: Google trends. Own elaboration.

In Fig 3, a daily average growth rate of the economic activity index of -33% can be seen for the second economic activity index, showing a great negative impact of the COVID-19 Pandemic. The so-called *pandemic economy* is quite unusual, it has identifiable characteristics and operates according to certain patterns. In the first stage, the outbreak must be contained at the expense of the strategy of suppression, fall in economic activity, loss of employment, reduction of consumption, and an economic recovery that cannot be specified as its end. This recovery will be much slower than the free-fall that occurs when locks are imposed according to the evidence [38].

As of May and June, it recovers more due to a rebound effect in the economic activity variable, but, above all, is explained by the economic policy and public health measures, the opening of the economic activation implemented by the Government. In Fig 2, economic activity would recover by up to 9.3% for May-June explained by the recovery in consumption that suggests a recovery to 75% of economic activity compared to the beginning of the pandemic.

In Fig 3, economic activity would fall at a lower rate of -9.8% for the economic activity indicator. Explained by a slow recovery due to investment expectations, increased unemployment, income inequality, and structural failures that suggests a recovery of 67% of economic activity compared to the beginning of the pandemic.

In Table 1, it shows the number of confirmed COVID-19 cases (*cvp*) reaching 2.090 per day for the study period. However, in the week of May 25, 2020, the maximum level of COVID-19 prevalence cases of 8.805 was reached, explained in part by the strategy of gradual opening in the reactivation of economic activities dictated by the Government. In the analysis period, it was recorded that the basic daily average spread index (*R*_*e*_) was 2.6, implying that a person can infect 2.6 susceptible people on average. The indicator shows a downward trend in the number of infected, but it still does not fall below unity, which would reflect that the infection would stop and, therefore, the Pandemic would end.

**Table 1:** Descriptive statistics of the variables of economic activity and determining factors. Daily data for the period 06/03/2020 to 07/06/2020.

Detail	Ye	Yi	cvp	dead	Re	td	prut	i	e	igb	cob	tem
Mean	71,4	53,5	2 090,4	67,5	2,6	10,6	12 680,6	0,7	2,5	82,2	92,3	22,1
Maxim	101,0	111,0	8 805,0	195,0	5,2	33,7	49 091,0	2,2	2,7	100,0	100,0	26,5
Minim	59,0	20,0	1,0	0,0	1,0	2,3	28,0	0,2	2,2	74,0	83,0	17,0
Standard deviation.	10,5	15,6	2 091,7	53,4	1,2	8,8	11 859,8	0,7	0,2	5,8	4,2	2,5
**Coefficient of variation**	**14,7**	**29,1**	**100,1**	**79,2**	**44,6**	**83,2**	**93,5**	**98,5**	**6,5**	**7,0**	**4,6**	**11,4**
Observations	94	94	94	81	94	94	94	94	94	94	94	94

Source: SENAMHI, MINSA, and COES. Own elaboration. NOTE: Ye, energy demand index in KW; Yi, index Google search on investment and employment; cvp, confirmed cases of COVID-19; dead, death cases from COVID-19; Re, indicates the basic propagation number for COVID-19; td, Case duplication time; prut, total samples; i, real interest rate; e, real exchange rate; igb, index of the Lima stock exchange; cob, international copper price index; and tem, the average temperature in°C.

More than 68 deaths/day (*dead*) have also been found on average, since the beginning of the COVID-19 Pandemic, with an indicator of doubling time (*T*_*d*_) of 10 days on average. Public spending on rapid, molecular, and serological tests (*pru*) has been limited to target and diagnose infected people, isolate and proceed with a timely health care protocol. The rest of the variables are control variables that affect the supply side and the aggregate demand.

**Fig 4:**
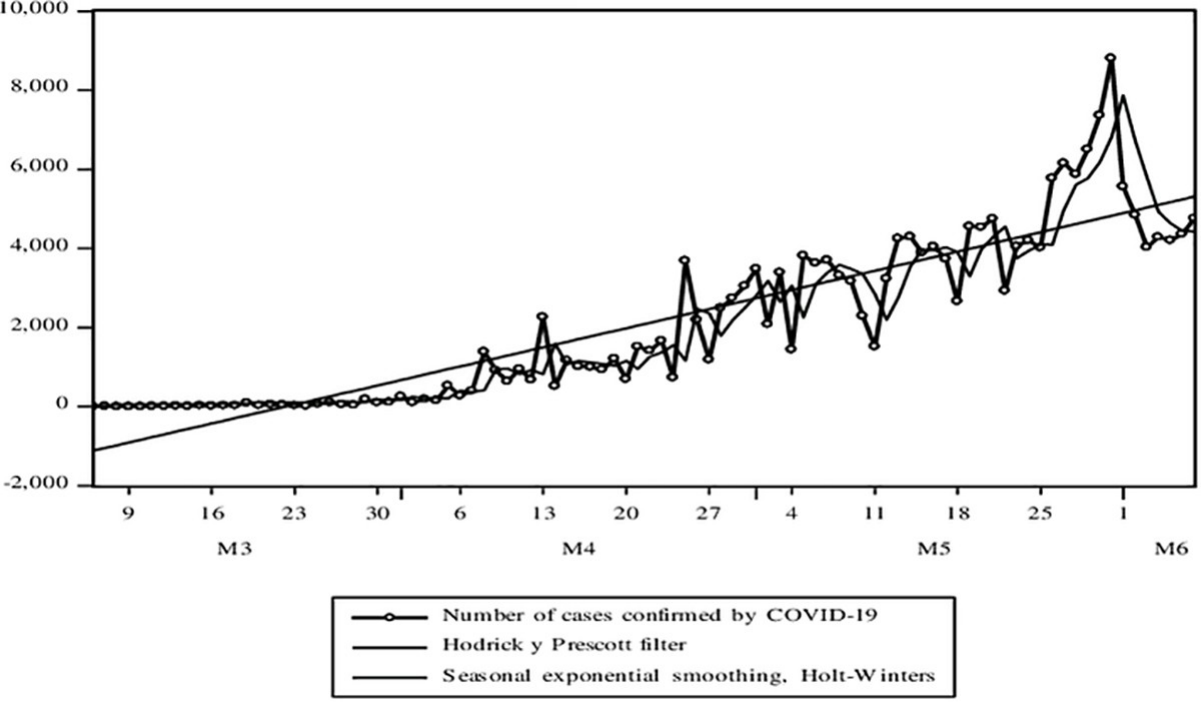
Evolution of time series associated with the COVID-19 pandemic. Confirmed cases, deaths, the basic number of spread, and the doubling time of cases. Source: MINSA. Own elaboration.

**Fig 5:**
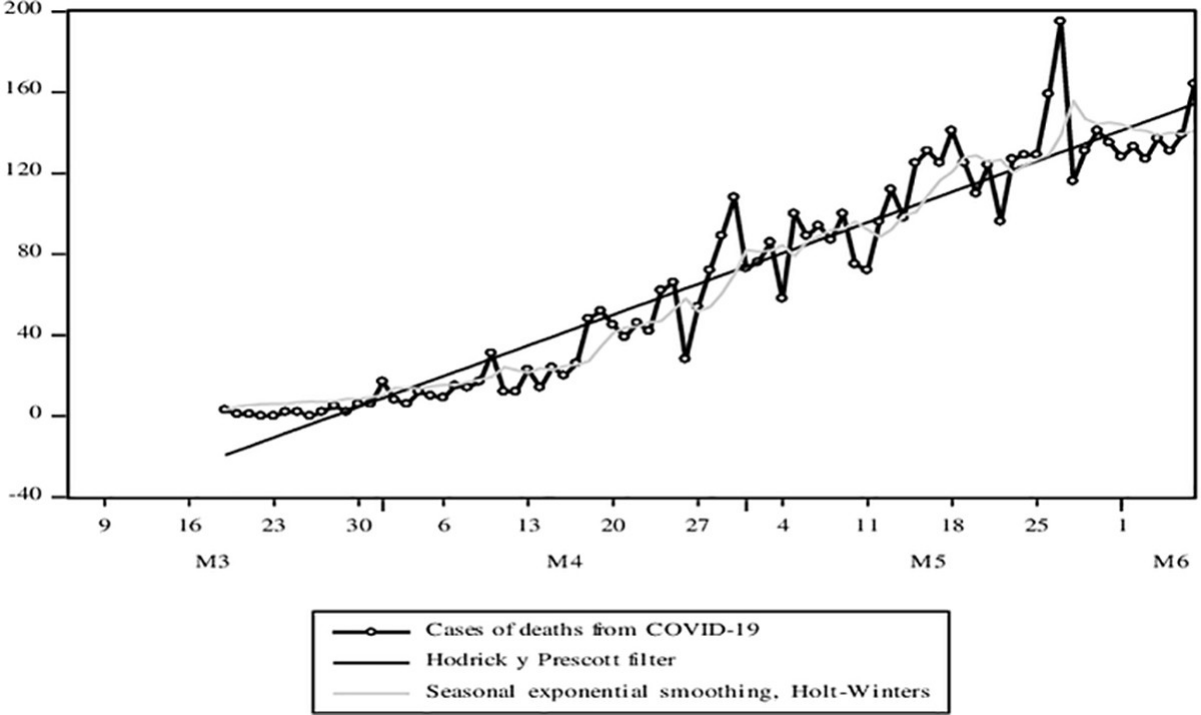
Evolution of time series associated with the COVID-19 pandemic. Confirmed cases, deaths, the basic number of spread, and the doubling time of cases. Source: MINSA. Own elaboration.

Here are some indicators of the Pandemic. Fig 4 shows the evolution of the variable that reflects the confirmed cases of COVID-19. The variable shows a behavior with an increasing and cyclical trend throughout the analysis period. And this cycle is volatile. The average growth rate according to the adjusted trend model would be 8.4%, that is, the confirmed cases of COVID-19 weekly on average have doubled. In Fig 5, the cases of deaths from COVID-19 show behavior with an increasing, cyclical, and volatile trend (less compared to the cases of infections). The growth rate of COVID-19 death cases according to the adjusted trend model resulted in 5.5%.

**Fig 6:**
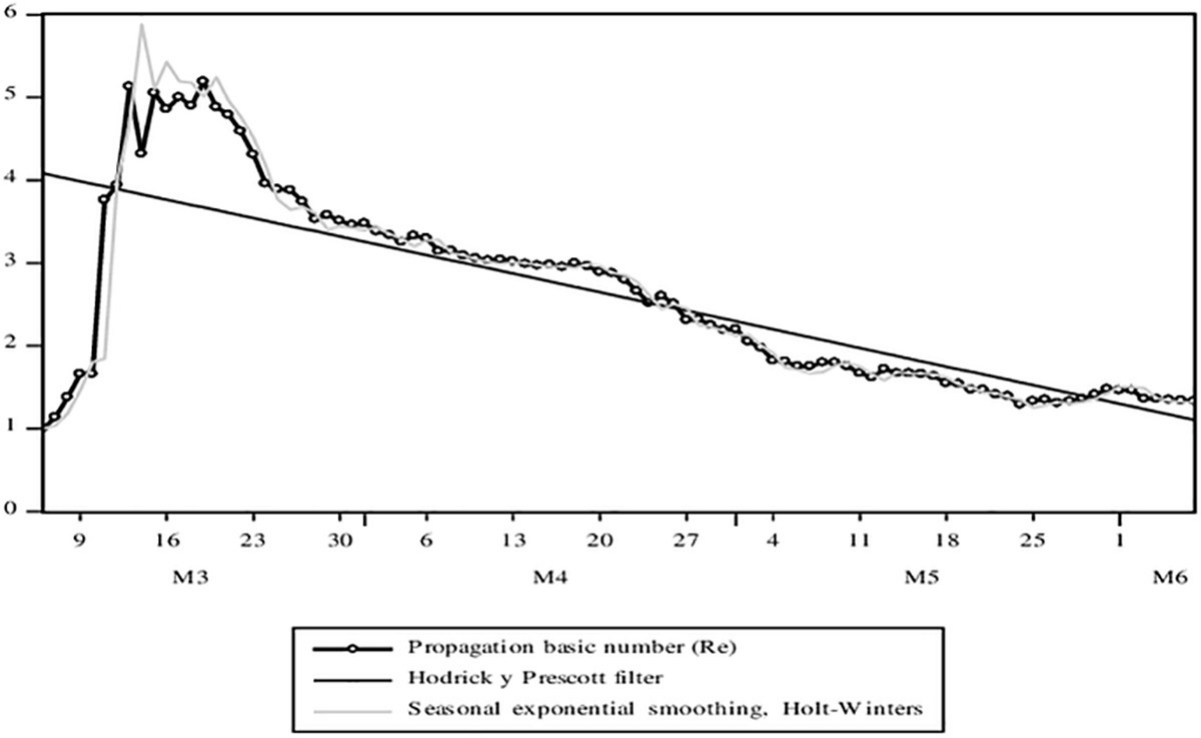
Evolution of time series associated with the COVID-19 pandemic. Confirmed cases, deaths, the basic number of spread, and the doubling time of cases. Source: MINSA. Own elaboration.

**Fig 7:**
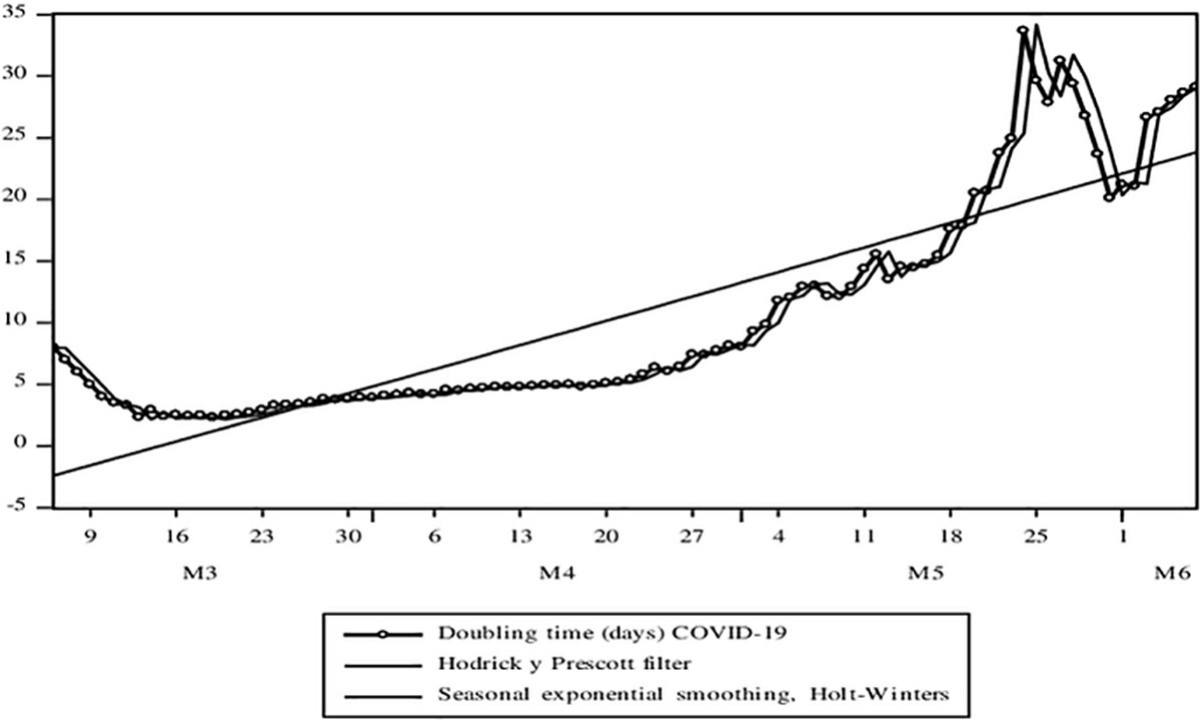
Evolution of time series associated with the COVID-19 pandemic. Confirmed cases, deaths, the basic number of spread, and the doubling time of cases. Source: MINSA. Own elaboration.

From the beginning of the Pandemic, according to the theoretical-mathematical model, two very relevant parameters can be obtained: the basic number of propagation (*R*_*e*_) and the doubling time of the cases (*T*_*d*_). First, in Fig 6, an initial R_e_ close to 6 has been obtained from which it has only gradually fallen, reaching the level of 1.7 on June 7, as the model suggests for the Peruvian case. However, serious structural limitations and the implementation of public policy measures have prevented this parameter from falling below 1, as suggested [34]. The average growth rate of R_e_ according to the adjusted trend model is 0.3%. Second, in Fig 7, T_d_ has a growth rate according to the 1.5% adjusted trend model for the period applying a logarithmic model of the variable as a function of time.

**Fig 8:**
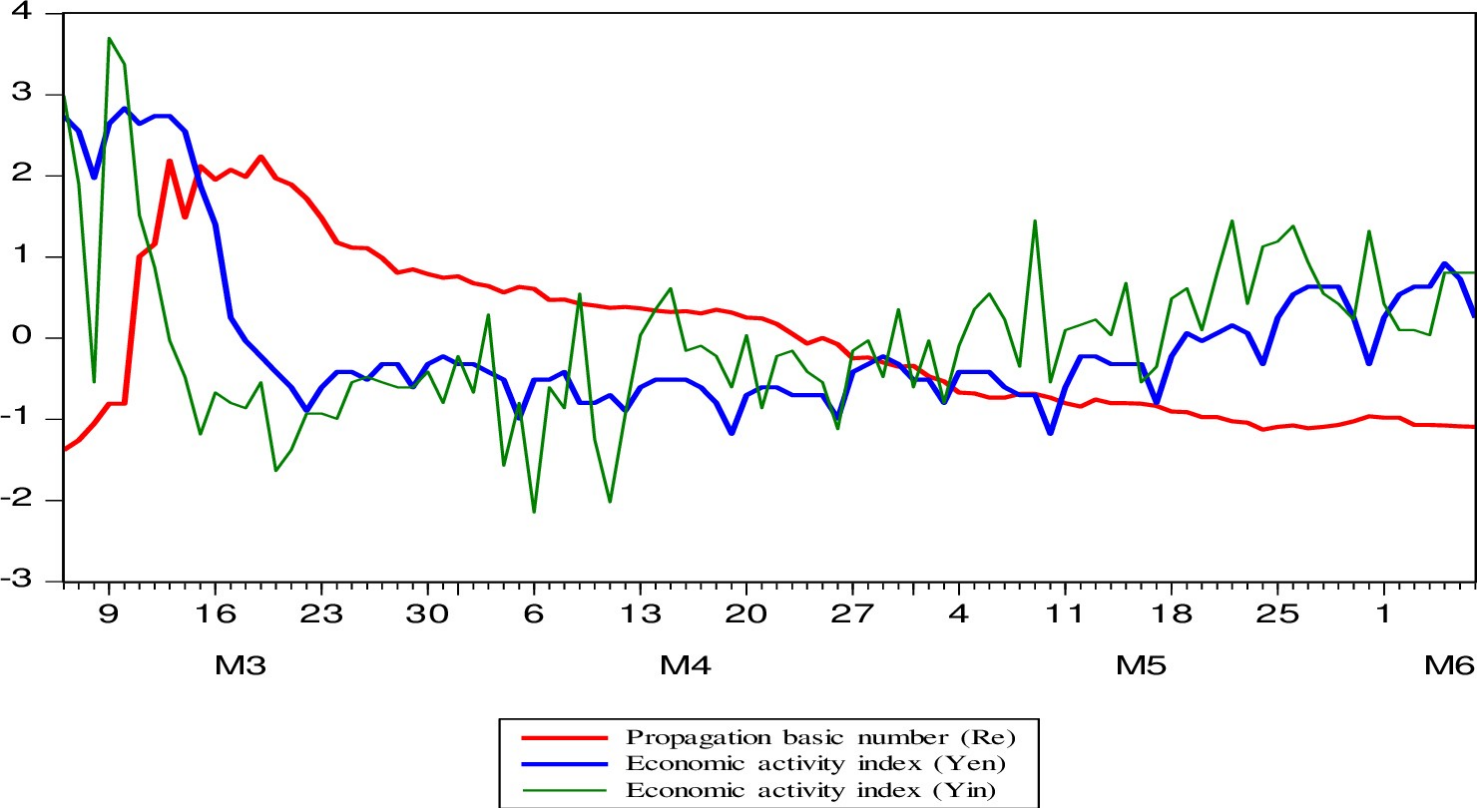
Evolution of economic activity (Yen and Yin) and the basic number of spread (Re). In index and normalized with the base date March 6 = 100. Analysis period from the start of the pandemic March 6 to June 7, 2020. Source: MINSA, COES, and Google. Own elaboration. n indicates that the series has been normalized.

**Fig 9:**
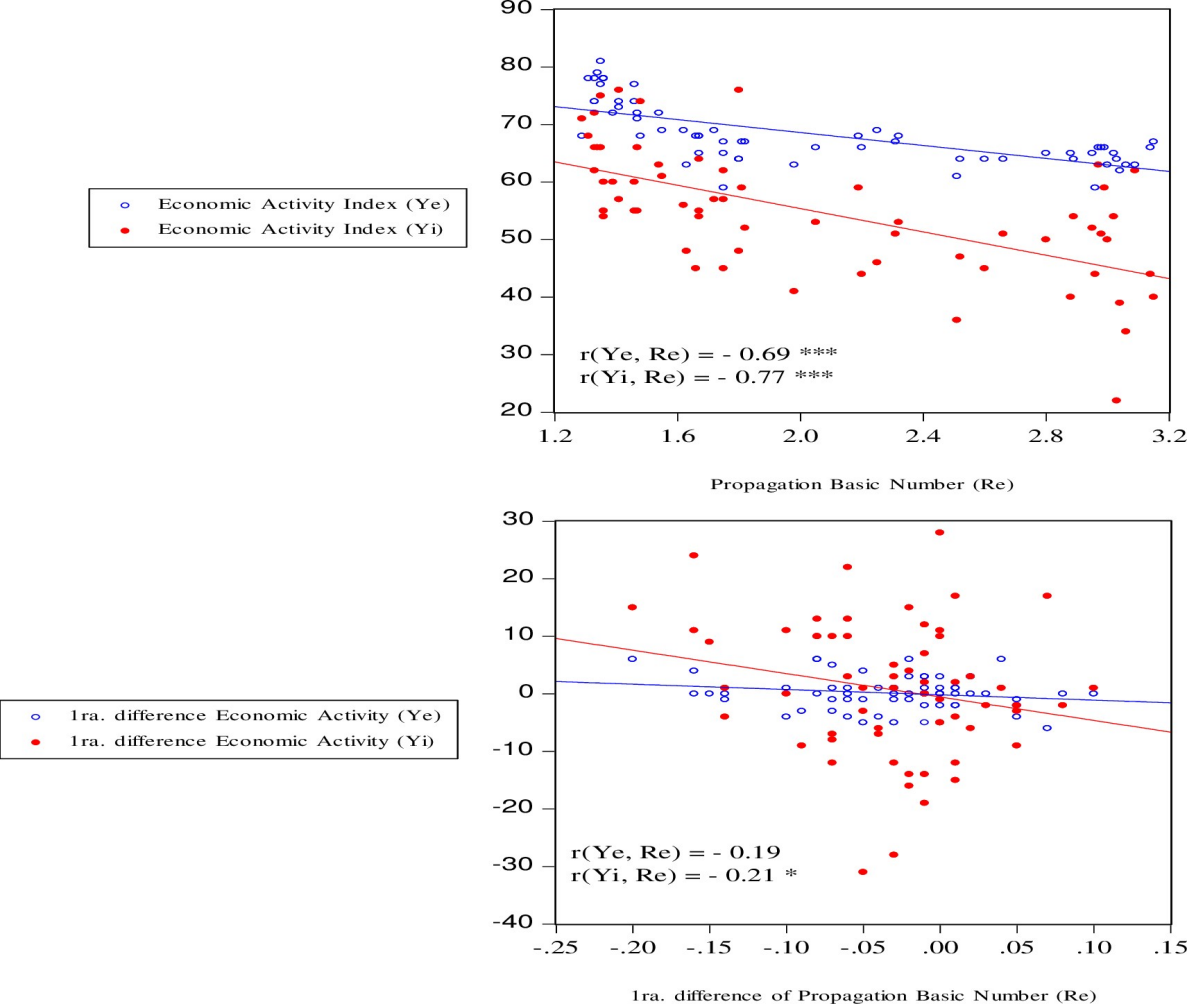
Dispersion and correlation propagation basic number of COVID-19 and economic activity (level and growth rate). Source: Own elaboration. NOTE: Ye and Yi have been chosen as indicators of economic activity. ***, **, * significant at 1%, 5%, and 10%. For n = 62; for n = 94 the results are less satisfactory for the first weeks that are volatile, typical of a Pandemic.

About the relationship between COVID-19 and economic activity. In Fig 8 it can be seen that both variables go together and there is evidence that graphically shows that there is a negative long-term relationship. Then, an analysis of the dispersion and correlation is presented in Fig 9, the negative correlation between the explanatory variable (R_e_) of the prevalence of COVID-19 with economic activity (Y_e_, Y_i_) is confirmed. Then, in terms of levels, economic activity is negative and statistically significant at 1% (r > -0.69), which reflects as a stylized fact for the short-term Peruvian economy that there is a negative association that reflects that when R_e_ increases, the level also falls. of economic activity and vice versa. In growth rates, the relationship is negative and significant at 10% (r > -0.21).

There is a negative relationship between the COVID-19 event and economic activity. But regardless of the theoretical models and empirical evidence, we infer that the Pandemic has shown a short vision of the future in our country. A review of what we want as a country is necessary, allowing the inclusion of the least favored or the informal workforce and reflecting weak governance, in a country that is dual, with oligopolistic power groups, with information and access privileged in public policies and public resources. A new social contract is necessary that favors the balance between private enterprise, civil society, and the State. In the short-term, an economy with resilience is reactivated, generating employment and income, and achieving inclusive and sustainable economic growth in the long-term.

### Covid-19 in Latin America

For Latin America, the IMF adjusts its economic growth forecast for 2020 by -9.4% and for Peru by -13.9%. About other economies in the region, the drop in economic activity would be one of the largest compared to Mexico (-10.5%), Brazil (-9.1%), Chile (-7.5%), Argentina (-9.9%), and Colombia (-7.8%) [7]. The Peruvian economy already showed slow and volatile economic growth, dependent on international markets and weak governance, with an annual average per capita GDP growth rate of 2% [58]; It also showed structural problems of economic duality, poverty, unequal income distribution [59], market failures, State failures, and failures of civil society [34]. The presence of COVID-19 exacerbated not only health but also economic and social inequality at the global level [37].

In Table 2 we show a summary of public health policies for 20 countries in Latin America. Overall we find three policies in response to COVID-19. A suppression policy, a mitigation policy, and a free policy (let the market work as is the case in the US) [60]. Countries like Chile, Brazil, Panama, Canada, and Peru have higher levels of infected per million. Countries like Peru, Chile, Canada, and Brazil have higher levels of deaths per million. Note that Peru reaches more than 576 deaths for every million inhabitants. Being the highest mortality rate in the world (all of them in America). For CEPAL, economic growth for the first half of 2020 affects countries such as Ecuador (-6.3%), Argentina (-5.7%), and Peru (-3.4%) to a greater extent. The forecast for 2020 is estimated to be a drop for Peru (-13%), followed by Argentina (-10.5%) and Brazil (-9.2%) [61, 62].

**Table 2:** Comparison of health policies for Latin America.

Latin American countries	Public Health Policies and Containment Measures	Health Impact (Infected per Million)	Health Impact (Deaths per Million)	GDP Growth rate I Sem 2020%	GDP Growth rate 2020 (CEPAL)
Argentina	A, B, C, D	3.960	73	-5.7	-10.5
Bolivia	A, B, D, E	6.445	247	-2.9	-5.2
Brazil	B, F, K	12.279	429	-1.5	-9.2
Chile	A, B, G, H	18.494	490	0.4	-7.9
Colombia	A, B, D, H, J, K	5.621	192	1.1	-5.6-
Costa Rica	B, D, G, H	3.394	27	-	
Ecuador	A, B, D, G, J	4.782	320	-6.3	-9.0
El Salvador	A, B, D, H, J	2.502	67	-	-
Guatemala	A, B, D, G, K	2.725	104	-	-
Honduras	A, B, G, D	4.182	132	-	-
Mexico	A, B, D, H, K	3.227	356	-1.6	-9.0
Nicaragua	A	554	17	-	-
Panama	A, B, D, G, J	14.877	323	-2.0	-
Paraguay	A, B, D	730	6	-1.0	-
**Peru**	**A, B, C, D, G, H, J, K, L**	**12.358**	**576**	**-3.4**	**-13.0**
Puerto Rico	Suppression politics	5.792	74	-	-
Dominican Republic	A, B, C, D, G	6.260	105	-	-
Uruguay	B, D	357	10	-3.0	-
US	Free politics	3.067	236	-1.3	-3.9
Canada	Mitigation politics	13.579	459	-2.1	-6.2

Source: IDB, World Bank, and ECLAC. Own elaboration. NOTE: In some countries, it has not been possible to access official information. Cuba and Venezuela are excluded due to access to information. NOTE: The nomenclature of public health policies and instruments is as follows: A: mandatory national confinement (quarantine); B: closing of schools, universities, commercial establishments, cinemas, theaters, and public places of massive attendance; C: mainly excluded from closing: supermarkets, food stores, pharmacies, notaries and banks; D: border closure; E: use of electronic anklets and handcuffs to follow up on diagnosed or suspected persons; F: only Brazilian nationals and residents are allowed to enter by air; G: national curfew; H: isolation for vulnerable people over 65 years of age, obese, hypertensive and diabetic; J: public transport operates in reduced capacity; K: restriction on the internal displacement of persons; L remote working.

## 2. Methods

To investigate the relationship between COVID-19 and economic activity, we used the ARDL model. We follow the new approach suggested [12, 63, 64], with a relationship between variables in levels that is applicable regardless if the underlying regressors are purely I (0), purely I (1), or mutually cointegrated. The statistic underlying our procedure is the Wald test or F statistic in a generalized Dicky-Fuller regression used to test the significance of lagged levels of the variables considered in an Error Correction (ECM) conditional equilibrium model. Additionally, Narayan tabulates critical values for sample sizes ranging from 30 to 80 observations, which are relevant to our research using limit tests with the cointegration approach [11, 65]. In contrast, Pesaran uses a sample of 1.000 [12]. This methodology allows considering the pandemic as an exogenous variable that explains economic activity, and considers other structural variables, but with the advantage of being a daily event with small samples.

The operational variables were selected according to availability and we considered a causal relationship proposed by economic science. In addition, it was sought that the information is available, daily, and complete. The sample size was considered from three months (n = 94 and n = 62). The F test allows us to verify the existence of significant statistics. For Narayan, the estimates of the ARDL method of cointegration analysis are unbiased and efficient since: a) it can be applied to studies that have a small sample, such as the present study; b) estimates the long- and short-term components of the model simultaneously, eliminating the problems associated with omitted variables and autocorrelation; and c) the ARDL model can distinguish between dependent and independent variables or endogenous and exogenous [11].

As the first stage in the methodology, the theoretical-mathematical model (S1 Model), was elaborated, to then select the available information: source, availability, and way of calculating (S1 Dataset), elaborate the most opportune stylized facts and ask ourselves the question investigation of the existence of an inverse relationship between COVID-19 and economic activity. The second stage consists of choosing the econometric model and determining the short and long-term relationship of COVID-19 with economic activity. For this, it was necessary to adjust the time series, apply a logarithm and normalize to be able to analyze it properly. It was necessary to carry out the economic, statistical, and econometric evaluation of the chosen optimal model. The third stage has been to prepare public policy recommendations to be considered and to answer the research question.

We present the theoretical functional form of the determinants of the level of economic activity and their expected causal relationships in Eq (1), with their operational variables. This equation is derived from the support information (S1 Model) in Eq (19).

y=f(Re,pru,i,e,cob,igb,tem,d1) (1)

Where:

$$\frac{\partial y}{\partial Re}<0,\frac{\partial y}{\partial pru}>0,\frac{\partial y}{\partial i}<0,\frac{\partial y}{\partial e}<>0,\frac{\partial y}{\partial cob}>0,\frac{\partial y}{\partial igb}>0,\frac{\partial y}{\partial tem}<0,\frac{\partial y}{\partial d1}<0$$

Then, the functional form of the double log-linear model using operational variables is specified. An empirical model with the economic activity variable in Eqs (2) and (3) as follows:

$$\begin{array}{l} ln{\left(Ye\right)}_{t}=\beta_{0}+\beta_{1}ln{\left(R_{e}\right)}_{t}+\beta_{2}\ln{\left(pru\right)}_{t}+\beta_{3}\ln{\left(i\right)}_{t}+\beta_{4}\ln{\left(e\right)}_{t}\\ \;+\beta_{5}\ln{\left(cob\right)}_{t}+\beta_{6}ln{\left(igb\right)}_{t}+\beta_{7}ln{\left(tem\right)}_{t}+\beta_{8}{d1}_{t}+\varepsilon_{t} \end{array}$$ (2)

$$\begin{array}{l} \ln{\left(Yi\right)}_{t}=\beta_{0}+\beta_{1}ln{\left(R_{e}\right)}_{t}+\beta_{2}ln{\left(pru\right)}_{t}+\beta_{3}ln{\left(i\right)}_{t}+\beta_{4}ln{\left(e\right)}_{t}\\ \;+\beta_{5}ln{\left(cob\right)}_{t}+\beta_{6}ln{\left(igb\right)}_{t}+\beta_{7}ln{\left(tem\right)}_{t}+\beta_{8}{d1}_{t}+\varepsilon_{t} \end{array}$$ (3)

Where:

ln (*Y*)_*t*_ is the logarithm of the index of economic activity per day. *Ye* is an operational variable of the level of economic activity, measured as the energy demand index and, *Yi* is also considered as an operational variable of the level of economic activity, measured as the mobility index.

ln(*R_e_*)_*t*_ is the logarithm of the index of the propagation basic number of COVID-19 per day.

*ln*(*prut*)_*t*_ is the logarithm of the current public expenditure index operationalized by the total diagnostic tests applied by COVID-19.

ln(*i*)_*t*_ is the logarithm of the reference real interest rate index per day.

ln(*e*)_*t*_ is the logarithm of the real exchange rate index per day.

ln(*cob*)_*t*_ is the logarithm of the international copper price index per day.

ln(*igb*)_*t*_ is the logarithm of the stock price index of the Lima Stock Exchange per day.

ln(*tem*)_*t*_ is the logarithm of the average temperature index per day.

*d*1_*t*_ is a *dummy variable* that captures the behavior of the strategy of suppression and opening of the economic activity implemented by the Government (0 when there is a suppression strategy and, 1 when the economic activity reopens in stages).

*ε_t_* Is the error term. Then *β*_1_,*β*_2_,*β*_3_…*β*_8_, are elasticities to be estimated.

The economic theory of exogenous and endogenous growth is considered as a reference. From the theories of exogenous growth we consider the seminal work of Solow [24, 25] and others [17–19], and on the theory of endogenous economic growth the work of Romer [22, 23] and others [16, 20, 21, 66]. The review of the literature and the mathematical model allows selecting the dependent variable and the independent variables. The model defines the endogenous variables and the exogenous variables. With the daily GDP, no work was found, however, there are studies on the impact of COVID-19 at the country level [2].

As the first operational variable of economic activity, energy demand is considered. Energy demand is the consumption of electricity as an indicator of economic activity. An advantage of using electricity demand as a measure of capital use is that it cannot be easily stored; therefore, the flow of electricity in a production process that corresponds exactly to the amount used in it. There is empirical evidence of the use of this variable for six developed economies [67] and Peru [68].

The second operational variable of economic activity is mobility. It is made from the Google search for employment and investment. A weighted index is achieved as a productive factor of 45% and 55% respectively. Some works find a correlation between macroeconomic variables with Google trends and the search for the term recession [45], economic activity [46] and economic sentiment [47]. It has also been used for diseases such as MERS [48], Dengue [49, 69] and AIDS [50, 51].

As an exogenous variable of the COVID-19 pandemic, the basic spread number R_e_ is used based on the parameters and the methodology suggested for the basic SIR model [70–74] and adapted to the Peruvian case [34]. Peru is one of the main countries in deaths, according to the number of cases per million. It is hypothetically assumed that there is an inverse causal relationship between the basic spread number and economic activity, which is deduced from the aggregate supply and demand model. As long as R_e_ > 1 holds, there will be a pandemic and economic activity will be recessed [1]. A study was found on the impact of a type of pandemic such as influenza on economic activity [26].

Current public spending allows a higher level of economic activity and economic growth. In the current conjuncture of a pandemic economy, this variable becomes a variable that avoids the abrupt fall in economic activity, in the case of a suppression strategy. Public spending policies that imply leaving *indiscriminate confinement with massive and intelligent applications* of tests to protect the population, in such a way that economic openness can be allowed [37, 39]. The study uses the total tests as an operational variable of public spending as an operational variable. It is hypothetically assumed that there is an inverse causal relationship between public spending that makes it possible to recognize COVID-19 and, therefore, detect it and provide timely treatment.

The real benchmark interest rate affects investment and aggregate demand. But on the side of the aggregate supply, it also affects via credit. The BCRP has systematically lowered the monetary policy reference interest rate from 2.5 in February to 1.25 in March (the beginning of the quarantine) and then to 0.25 in April, remaining at an all-time low. It is hypothetically assumed that there is an inverse relationship between the real interest rate that is determined from the aggregate demand and supply model. Therefore, the reduction of said interest rate leads to an expansion of economic activity. Market liquidity was achieved with credit auctions [75], with the “*Reactiva Peru”* Program which in the first stage granted US$ 7.5 billion [40] and in the month of August in its second stage, it reached a total of US$ 16 billion [2]. Furthermore, the delivery of cash bonds directly to the vulnerable population leads to increasing consumption and partially reversing the deep recession. Also, there are other forms of proven monetary policy for developed countries, such as asset purchases, which do not necessarily apply to Peru [76].

The real exchange rate affects exports and, therefore, aggregate demand. On the one hand, it affects the domestic market with the loss of purchasing power and, on the other hand, it affects the trade balance [77]. The BCRP does not control the exchange rate, however, it systematically conducts open market operations to keep it in a band. Exchange intervention has been with greater emphasis in March and April [78]. It is hypothetically assumed that there is a direct or inverse causal relationship between the real exchange rate and economic activity, a causality that derives from the aggregate supply and demand model and the Mundell-Fleming model.

The international price of copper is determined by international markets, affects exports and aggregate demand. Peru is one of the main exporting countries of raw materials, especially minerals and these prices fell in March, but then they have systematically increased until July, due to the general increase in commodities [75]. It is assumed that there is a direct causal relationship between the price of copper and economic activity [10] and is derived from the aggregate supply and demand model.

The stock price index of the Lima Stock Exchange is hypothetically expected to have a positive causal relationship with economic activity, as derived from the aggregate supply and demand model. Only in August had it fallen -9.5% compared to July. And the evidence for emerging countries has been that COVID-19 has had a negative impact, both in developed and emerging countries [79].

It is hypothetically assumed that there is an inverse causal relationship between temperature and economic activity [27–31]. Climate is considered a relevant variable that is correlated with COVID-19 [80, 81], which increases the probability of risk of incidence and contagion [82] and indirectly affects economic activity. However, there are no definitive studies yet [83].

The variable *d*1_*t*_ is a dummy variable to capture the behavior of the application of the strategy of suppression and subsequent reopening of economic activity in stages, implemented by the Government [73, 84]. It is hypothetically assumed that there is an inverse causal relationship between the implementation of the suppression strategy and economic activity.

### ARDL model: Short-term and long-term elasticities

The Eqs (2) and (3) specified above are static and we want to analyze the dynamics of economic activity based on its lagged variable as well as the explanatory variables and their lags, for this, we use the ARDL methodology. We find a long-term relationship from Eqs (2) and (3). The ARDL model (p, q_1_, q_2_… q_7_), with a lag, the Eqs (4) and (5) remain as follows:

$$\begin{array}{l} ln{\left(Ye\right)}_{t}=\beta_{0}+\beta_{1}ln{\left(Ye\right)}_{t-1}+\beta_{2}ln{\left(R_{e}\right)}_{t}+\beta_{3}ln{\left(R_{e}\right)}_{t-1}+\beta_{4}\ln{\left(pru\right)}_{t}\\ \;+\beta_{5}\ln{\left(pru\right)}_{t-1}+\beta_{6}\ln{\left(i\right)}_{t}+\beta_{7}\ln{\left(i\right)}_{t-1}+\beta_{8}\ln{\left(e\right)}_{t}\\ \;+\beta_{9}\ln{\left(e\right)}_{t-1}+\beta_{10}\ln{\left(cob\right)}_{t}+\beta_{11}ln{\left(cob\right)}_{t-1}+\beta_{12}ln{\left(igb\right)}_{t}\\ \;+\beta_{13}ln{\left(igb\right)}_{t-1}+\beta_{14}ln{\left(tem\right)}_{t}+\beta_{15}ln{\left(tem\right)}_{t-1}+\beta_{16}{d1}_{t}\\ \;+\varepsilon_{t} \end{array}$$ (4)

$$\begin{array}{l} \ln{\left(Yi\right)}_{t}=\beta_{0}+\beta_{1}ln{\left(Yi\right)}_{t-1}+\beta_{2}ln{\left(R_{e}\right)}_{t}+\beta_{3}ln{\left(R_{e}\right)}_{t-1}+\beta_{4}\ln{\left(pru\right)}_{t}\\ \;+\beta_{5}\ln{\left(pru\right)}_{t-1}+\beta_{6}\ln{\left(i\right)}_{t}+\beta_{7}\ln{\left(i\right)}_{t-1}+\beta_{8}\ln{\left(e\right)}_{t}\\ \;+\beta_{9}\ln{\left(e\right)}_{t-1}+\beta_{10}\ln{\left(cob\right)}_{t}+\beta_{11}ln{\left(cob\right)}_{t-1}+\beta_{12}ln{\left(igb\right)}_{t}\\ \;+\beta_{13}ln{\left(igb\right)}_{t-1}+\beta_{14}ln{\left(tem\right)}_{t}+\beta_{15}ln{\left(tem\right)}_{t-1}+\beta_{16}{d1}_{t}\\ \;+\varepsilon_{t} \end{array}$$ (5)

Where, when applying the Bounds Test it is determined if the model cointegrate. That is, it is determined if there is a long-term relationship, which would lead us to Eqs (6) and (7) as follows:

$$\begin{array}{l} ln{\left(Ye\right)}_{t}=\beta_{0}+\sum_{i=1}^{p}\beta_{1}ln{\left(Ye\right)}_{t-i}+\sum_{i=0}^{q1}\beta_{2}ln{\left(R_{e}\right)}_{t-i}+\sum_{i=0}^{q2}\beta_{3}\ln{\left(pru\right)}_{t-i}\\ \;+\sum_{i=0}^{q3}\beta_{4}\ln{\left(i\right)}_{t-i}+\sum_{i=0}^{q4}\beta_{5}\ln{\left(e\right)}_{t-i}+\sum_{i=0}^{q5}\beta_{6}ln{\left(cob\right)}_{t-i}\\ \;+\sum_{i=0}^{q6}\beta_{7}ln{\left(igb\right)}_{t-i}+\sum_{i=0}^{q7}\beta_{8}ln{\left(tem\right)}_{t-i}+w_{t} \end{array}$$ (6)

$$\begin{array}{l} ln{\left(Yi\right)}_{t}=\beta_{0}+\sum_{i=1}^{p}\beta_{1}ln{\left(Yi\right)}_{t-i}+\sum_{i=0}^{q1}\beta_{2}ln{\left(R_{e}\right)}_{t-i}+\sum_{i=0}^{q2}\beta_{3}\ln{\left(pru\right)}_{t-i}\\ \;+\sum_{i=0}^{q3}\beta_{4}\ln{\left(i\right)}_{t-i}+\sum_{i=0}^{q4}\beta_{5}\ln{\left(e\right)}_{t-i}+\sum_{i=0}^{q5}\beta_{6}ln{\left(cob\right)}_{t-i}\\ \;+\sum_{i=0}^{q6}\beta_{7}ln{\left(igb\right)}_{t-i}+\sum_{i=0}^{q7}\beta_{8}ln{\left(tem\right)}_{t-i}+w_{t} \end{array}$$ (7)

The order of the lag is selected according to the Schwarz criterion as recommended by Pesaran and Shin [63, 85], who suggest models with a maximum of two lags.

### Error Correction Model (ECM)

In the presence of cointegration, the short-term elasticities can also be derived using an ECM in Eqs (8) and (9) as follows:

$$\begin{array}{l} \Delta ln{\left(Ye\right)}_{t}=\beta_{0}+\sum_{i=1}^{p}\beta_{1}\Delta ln{\left(Ye\right)}_{t-i}+\sum_{i=0}^{q1}\beta_{2}\Delta ln{\left(R_{e}\right)}_{t-i}+\sum_{i=0}^{q2}\beta_{3}\Delta \ln{\left(pru\right)}_{t-i}\\ \;+\sum_{i=0}^{q3}\beta_{4}\Delta \ln{\left(i\right)}_{t-i}+\sum_{i=0}^{q4}\beta_{5}\Delta \ln{\left(e\right)}_{t-i}+\sum_{i=0}^{q5}\beta_{6}\Delta ln{\left(cob\right)}_{t-i}\\ \;+\sum_{i=0}^{q6}\beta_{7}\Delta ln{\left(igb\right)}_{t-i}+\sum_{i=0}^{q7}\beta_{8}\Delta ln{\left(tem\right)}_{t-i}+\beta_{9}{d1}_{t}\\ \;+\psi {\left(ECM\right)}_{t-1}+\vartheta_{t} \end{array}$$ (8)

$$\begin{array}{l} \Delta ln{\left(Yi\right)}_{t}=\beta_{0}+\sum_{i=1}^{p}\beta_{1}\Delta ln{\left(Yi\right)}_{t-i}+\sum_{i=0}^{q1}\beta_{2}\Delta ln{\left(R_{e}\right)}_{t-i}+\sum_{i=0}^{q2}\beta_{3}\Delta \ln{\left(pru\right)}_{t-i}\\ \;+\sum_{i=0}^{q3}\beta_{4}\Delta \ln{\left(i\right)}_{t-i}+\sum_{i=0}^{q4}\beta_{5}\Delta \ln{\left(e\right)}_{t-i}+\sum_{i=0}^{q5}\beta_{6}\Delta ln{\left(cob\right)}_{t-i}\\ \;+\sum_{i=0}^{q6}\beta_{7}\Delta ln{\left(igb\right)}_{t-i}+\sum_{i=0}^{q7}\beta_{8}\Delta ln{\left(tem\right)}_{t-i}+\beta_{9}{d1}_{t}\\ \;+\psi {\left(ECM\right)}_{t-1}+\vartheta_{t} \end{array}$$ (9)

Where ECM_t-1_ is the error correction term (ECM). Δ is the first difference operator. β´s are the coefficients related to the short-term dynamics of the equilibrium convergence model. *ψ* is the measure of the adjustment speed. ARDL models are estimated by OLS with the endogenous variable as a function of the exogenous variables and with lags. The optimal combination is then established as the one that minimizes according to the Schwarz Criterion (SC).

The short-term analysis finds a long-term Cointegration relationship between the exogenous variables and the endogenous variable. It is tested if there is a long-term relationship between the variables, through the bounds test. An F-test is used with the null hypothesis that the variables are not cointegrated. The test statistic is computed and compared with two asymptotic critical values corresponding to cases where the variables are I (0) or I (1). When the test statistic is above the upper critical value, it rejects the null hypothesis (Ho) and concludes that cointegration is possible and an ECM model can be estimated.

We can mention as limitations: i) the availability of reliable macroeconomic variables [38], ii) limited access to *open data* [34], iii) the nature of the daily data considered that allows describing the trend and impact, and iv) the nature of the pandemic as a shock that affects both aggregate demand and aggregate supply. With this, v) the first 4 weeks the variable R_e_ is volatile, therefore a second model with (62 observations) is estimated as an alternative.

## 3. Results

**First**, it is found that the time series are stationary of order one I (1) with the unit root tests (S1 Annex) and there is causality between COVID-19 and economic activity, at 1% statistical significance (p = 0.00 <0.05 and p = 0.00 <0.05, respectively), with the Granger causality test. That is, the basic number of propagation Re, causes the level of economic activity. In S2 Annex, it can be seen that the test for the rest of the variables is most significant.

**Second,** there is evidence of existence of a long-term Cointegration relationship for the four models, according to the Bounds test, at a significance level of 1% with the F-test and t-test (Table 3). Given that the value of the F-statistic and the value of the t-statistic are greater than the critical values, the four models cointegrate in the long-term, by rejecting the null hypothesis that there is no long-term relationship.

**Table 3:** Cointegration with the Bounds test.

*Model*	*Variable*	*Bounds test*		*Cointegration*	*Process*
	*Dependent*	*F-statistic*	*t-statistic*	*long-term*	*ARDL or ECM *
*I*	*ln(Ye)*	*6*.*68>3*.23***	*-5*.*29>-3*.43***	*Yes*	*Estimate ECM*
*II*	*ln(Yi)*	*9*.*07>3*.23***	*-8*.*37>-3*.43***	*Yes*	*Estimate ECM*
*III*	*ln(Ye)*	*5*.*80>2*.96***	*-5*.*18>-3*.43***	*Yes*	*Estimate ECM*
*IV*	*ln(Yi)*	*8*.*05>3*.31***	*-6*.*76>-3*.43***	*Yes*	*Estimate ECM*

Own elaboration.

*, **, *** indicate statistical significance at 10%, 5% and 1% respectively. NOTE: Model I uses Eq (4); model II Eq (5) for n = 94 data. With n k = 7 regressors. Model III uses Eq (4) and model IV uses Eq (5), but with n = 62 data. The estimates use a model with constant and no trend.

If the value of the F-statistic is less than the critical values estimated for n = 94 and n = 62 of the sample size, the null hypothesis could not be rejected. Therefore, the best model would only be a difference model. However, given the results, we first estimate the level model and then an ECM model, according to the procedure [11, 12].

Then, if the Cointegration condition holds for both equations with a value of the F-statistic (F_Ye_ = 6.68 > 5.80) and (F_Yi_ = 9.07 > 8.05), according to the sample size (n = 94 vs n = 62), at the 1% significance, would imply that there is a single long-term equilibrium Cointegration relationship in both models.

**Third, 4 estimated models are shown according to Eqs (4**) **and (5**)**. The best ARDL model is model III. The ARDL model presents a good performance of 84%** (Table 4). Where the economic activity is explained by the lag in economic activity LogY_e_(-1) with the expected sign, which would confirm that the economic activity lagged in the previous days explains the levels of contemporary economic activity, causing ***a contagion effect*** and expanding the impact. **COVID-19, operationalized by the Propagation basic number Log (R**_**e**_**), has a negative sign and is statistically significant at 5%, corroborating our working hypothesis**. The lag in public spending on rapid, molecular and serological tests, Log(pru(-1)) has a negative sign and is statistically significant at 5% and; the dummy variable (d1) associated with the strategy of suppression and reopening of economic activity is positive at 1% of statistical significance, according to the expected causality.

**Table 4:** Estimation of the ARDL model.

Variable	Model I ARDL (1,0,0,0,0,0,0,0)	Model II ARDL (1,0,0,0,0,0,0,0)	Model III ARDL (1,0,1,0,0,0,0,0)	Model III ARDL (1,0,0,1,0,0,0,0)
**LOG(YE(-1))**	**.54**	---	.38***	---
** **	(6.44)	---	(3.22)	---
	[.00]	---	[.00]	---
**LOG(YG(-1))**	**---**	**.10**	---	**.09**
** **	**----**	(1.01)	---	(.71)
	**---**	[.31]	---	[.47]
**LOG(Re)**	**- .01**	- .30**	- .15**	**- .35**
** **	(- .34)	(- 2.29)	(- 2.25)	(- 1.07)
	[.73]	[.02]	[.02]	[.28]
**LOG(PRU)**	-.02**	**.02**	**-.01**	**-.01**
** **	(-2.15)	(.60)	(-.99)	(-.17)
	[.03]	[.54]	[.32]	[.86]
**LOG(PRU(-1))**	---	**---**	-.02**	---
** **	---	---	(-2.16)	---
	---	---	[.03]	---
**LOG(I)**	**.01**	**.03**	**- .02**	-.23*
** **	(.82)	(.40)	(.78)	(-1.71)
	[.41]	[.68]	[.43]	[.09]
**LOG(I(-1))**	**---**	**---**	**---**	.27**
** **	**---**	**---**	**---**	(2.34)
	**---**	**---**	**---**	[.02]
**LOG(E)**	-.08**	**-.40**	**-.54**	**.04**
** **	(-.62)	(-.51)	(-1.30)	(.02)
	[.53]	[.60]	[.19]	[.98]
**LOG(COB)**	.74**	**.92**	**.004**	**1.62**
** **	(3.08)	(.78)	(.01)	(.79)
	[.00]	[.43]	[.99]	[.43]
**LOG(IGB)**	**-.16**	**.72**	**-.10**	**1.02**
** **	(-.91)	(.75)	(-.31)	(.62)
	[.36]	[.45]	[.75]	[.53]
**LOG(TEM)**	-.13*	**.37**	**-.14**	**.50**
** **	(-1.64)	(.83)	(-1.36)	(.93)
	[.10]	[.40]	[.18]	[.35]
**D1**	.52***	**.03**	.05***	**-.05**
** **	(3.57)	(.44)	(2.98)	(-.57)
	[.00]	[.66]	[.00]	[.56]
**C**	**.32**	**-3.68**	6.71**	**-10.44**
	(.27)	(-.59)	(2.62)	(-.76)
	[.78]	[.55]	[.01]	[.44]
**R-squared**	**0.92**	**0.50**	**0.84**	**0.47**
Maximum dependent lags (A*utomatic selection*)	1	1	1	1
Model selection method (*Schwarz criterion- SC*)	-3.40	0.09	-3.38	-.12
Daily data (*07/03-07/06/2020*)	94	94	62	62
Durbin-Watson	1.85	2.06	1.80	1.80
F	116.95***	9.10***	25.91***	4.45***
**Breusch-Godfrey Serial Correlate LM Test**				
. Prob F(2,81) and F(2,49) respectively	0.84	0.42	0.55	0.19
. Accept Ho: Residues no correlate				
**Breusch-Pagan-Godfrey Test**				
. Prob F(9,83) and F(10, 51) respectively	0.70	0.03	0.66	0.72
. Rejection Ho: Residues are homoscedastic				
**RESET—Ramsey Test (Fs)**				
. Prob Fs (1,82) and (1,50) respectively	7.24 [.01]	5.96 [.02]	0.26** [.61]	5.78 [.02]
. Accept Ho: Correct functional form				

Source: Own elaboration. NOTE: Model I uses Eq (4); Model II Eq (5) for n = 94 data. With k = 7 regressors. Model III uses Eq (4) and Model IV uses Eq (5), but with n = 62 data. LOG, indicates the logarithm of the time series. (), indicates t-statistic y [], probability.

*, **, *** Indicate statistical significance at 10%, 5%, and 1% respectively.

The rest of the exogenous variables, such as the real interest rate, the real exchange rate, and the stock price index of the stock market and temperature, show an expected negative sign but are not statistically significant. The price of copper also meets the expected positive sign, but it is not statistically significant.

Endogeneity problems arise when there is a correlation between exogenous variables and model error. It can be explained by measurement errors, autocorrelation, simultaneity, and omitted variables. Econometric evaluations for the first three problems are justified. However, this is not the case for omitted variables [86, 87]. Using n = 94 data for Model I the RESET statistic is 7.24; this value of the random variable F_(1,82)_ and its associated p-valor is 0.0082. This detects a poor functional specification. For model II, the RESET statistic is 5.96 for the random variable F_(1,82)_ and its p-valor associated is 0.016.

Using n = 62 data for Model III the RESET statistic is 0.262; this value of the random variable F_(1,50)_ and its p-valor associated is 0.6108. This detects a good functional specification. For model IV, the RESET statistic is 5.779 for the random variable F_(1,50)_ and its associated p-value is 0.020. Therefore, we do not reject the 5% level of significance.

Based on the RESET statistic, we chose model III with n = 62 data. The residuals of model III are estimated with n = 62 and included in the model with all the explanatory variables. It was obtained that the statistical t of the residuals is t = 1.043 with the associated p-value of 0.301, which corroborates that the endogeneity problem does not exist [86, 87].

The chosen model passes the tests of the Gauss-Markov Theorem, multicollinearity, significance of the serial correlation, and the homoscedasticity test in a significant way. The choice of Model III is confirmed that passes the model specification and stability test. From the ARDL III model, the ECM Model V is estimated according to the econometric evaluation because it has the expected coefficient and is statistically significant.

**Fourth**, the short-term ECM model V is estimated in the first differences. This model is estimated from model III ARDL. In the short-term, economic activity is explained to a greater extent by the variable of current public spending, contemporary total diagnostic tests (pru), but it is not statistically significant.

Then, the contemporary *dummy variable* (d1) has a positive and statistically significant short-term impact at 1% in the contemporary period and refers to the strategy of suppression and reopening of economic activity in stages. For the short-term model, errors lagged by one ECM(-1) meet the expected negative sign and are statistically significant. The value obtained of -0.61, implies that the model variables reach an equilibrium in the long-term (Cointegration) and that in the short-term they are corrected at a daily adjustment speed of 61% (Table 5).

**Table 5:** Estimation of the ECM (model V).

*Variable*	*Model V ARDL-CEM (1*,*0*,*1*,*0*,*0*,*0*,*0*,*0)*
*Dlog(pru)*	***-*.*01***
	*(-1*.*57)*
	*[*.*12*]
*C*	*6*.71***
	*(7*.*26)*
	*[*.*00*]
*d1*	.05***
	*(4*.*76)*
	*[*.*00*]
***ECM(-1)***	*-*.61***
	*(-7*.*26)*
	*[*.*00*]
***R***^***2***^	**.*48***
***Schwarz***	*-3*.*85*
***F***	*17*.97***
***n***	*62*

Source: Own elaboration. (), indicates t-statistic y [], probability.

*, **, *** Indicate statistical significance at 10%, 5%, and 1% respectively. D, first difference.

**Lastly,** the long-term elasticity analysis of the V model is presented. It indicates that the Propagation basic number of COVID-19 is negative and statistically significant at 5% with a coefficient of -0.24; that the current public expenditure measured with the total tests is negative with a coefficient of -0.05, statistically significant at 10%. The rest comply with the sign but are not statistically significant (Table 6).

**Table 6:** Long-term model (with elasticities).

Variable	Model V
LRe	-.24**
	(2.07)
	[.04]
Lpru	-.05*
	(1.99)
	[.05]
Li	-.03
	(-.75)
	[.45]
Le	-.88
	(1.30)
	[.20]
Lcob	.01
	(.01)
	[.99]
Ligb	-.16
	(-.30)
	[.75]
Ltem	-.22
	(1.37)
	[.17]

Source. Own elaboration. NOTE: The long-term elasticities of each model are shown. L indicates a logarithm.

It is concluded, that the ARDL model is relevant to explain the impact of COVID-19 on economic activity. The *contagion effect* with lagged economic activity in a period of one day is tested, public spending has had a significant negative impact on contemporary economic activity and that the Propagation basic number (Re) that reflects the shocks of aggregate supply and aggregate demand, has a negative and statistically significant impact at 5% on the level of contemporary economic activity. Also, there is empirical evidence, which supports, according to the ECM, that the strategy of suppression and the gradual opening of economic activities has a positive impact on the economic growth rate. It implies that said public policy of gradually eliminating suppression (quarantine) and opening economic activity in stages, implies gradually reducing the economic recession, but at the cost of continuing to increase the number of infected and deaths, reflected in a Re> 1.

## 4. Discussion

### 4.1 Empirical evidence is found through the ARDL model and the Granger test that systemic shocks (COVID-19) explain the recession in economic activity

The best model that explains economic growth is model V (ECM) explained by the growth of public spending, the openness policy, and the ECM, which adjusts at a rate of 61% per day.

### 4.2 The COVID-19 shock has an inverse impact on economic activity in Peru

With an elasticity of -0.15 in the short-term and -0.24 in the long-term, both statistically significant at 5%. This implies that the 1% decrease in R_e_ implies that economic activity would increase by 15% in the short-term and 24% in the long-term. It is recommended to continue with the policies to detect, target, and eradicate COVID-19 with the gradual opening of economic activity and with unlimited diagnostic tests, prioritizing the detection of the virus in risk areas or activities with massive human groups, with health policies preventive measures complemented with behavioral economics policies that encourage the use of masks, hand washing and respect for social distancing.

There is a trade-off between life and economic reactivation, as it tends to prioritize the reduction of R_e_ below unity. The Government has taken the steps for economic opening and co-movements are taking place in the model [3–6]. With this, it is necessary to implement policies for the reactivation of aggregate demand and aggregate supply in the short, medium, and long term as an alternative [5, 35–37]. We recognize that there is an impact on the side of the external and internal shock, but this is a structural characteristic of the Peruvian economy that is not the object of this investigation.

### 4.3 Public spending has an inverse impact on economic activity and is statistically significant at 5% with a lag, with a short-term elasticity of -2.0% and a long-term elasticity of -5.0%, statistically significant at 10%

The sign is not as expected following the positive Keynesian causality of public spending, which could be explained by the structural characteristics and institutional weakness reflected by an ineffective and inopportune implementation of the aforementioned public health policy. Also, very limited coverage, which reflects inefficient management of current public spending in particular and of the public apparatus in general, by the Government accompanied by the corruption of public officials. This also reflects the scarce investment in the training and protection of human capital qualified in the specialty and equipped, which has not allowed a rapid, timely, and effective response to face the pandemic.

**A higher allocation of current public spending is recommended for the acquisition and unlimited use of rapid, molecular, and serological tests that detect the direct presence of the COVID-19 virus, and recommended for the evaluation of interventions** [37]. This is done in a public-private alliance for investment in recognized laboratories, private clinics, public-private universities, and the armed forces for the production of medicines and access to the vaccine; countries like South Korea and Vietnam already market their intangibles and equipment for viral diseases. In the long term, the evolution of new viruses will continue and a plan is needed that considers the health economy, behavioral economy, and knowledge economy, seeking to implement incentive policies especially for the informal labor sector, complemented with inclusion policies considering norms and customs.

The experimental economy can help people to modify their behavior about Public Health Policies in terms of sanitary measures, social distancing and promote incentives for entrepreneurship, business creation, savings, investment, and creativity, achieving encourage *the production and provision of public and social goods and services* from small, informal or low-income businesses because the private sector and the market do not prioritize it. That it be the State that orders and organizes what should be produced and that it be the private sector that responds creatively or innovating in public and private goods and services and that satisfy said demand with domestic and non-imported products, tending to reverse the deep recession.

### 4.4 The policy of suppression and quarantine imposed by the Government of Peru has a negative impact on economic activity, but after the gradual and staggered re-opening of economic activities in stages, as of May 25, the impact is gradually reversed, being positive and statistically significant at 5%

The suppression strategy was not well received by the population, especially by the informal sector, but it was established as the only way to reduce the curve of infected cases, and obeying a Law. This is one of the quarantines of more long duration compared to other countries. And after three months, the policy of suppression has affected the economy of Peruvian households (more than 70% of the population belongs to an informal labor market). The population is forced to mobilize to work and obtain their low income, which is intended for consumption and not for saving (subsistence income). Other households had to go into debt to consume and face the Pandemic [34].

The suppression policy not only implied flattening the curve of those infected by COVID-19, reducing R_e_, but it is associated with the cases of infections and deaths that are increasing, and that is related to an economic curve of recession or fall in the level of economic activity in the short-term and the trend towards a decline in economic growth in the long-term [2, 10]. It is recommended to change from an indiscriminate suppression policy strategy to a selective, focused, and intelligent mitigation strategy that minimizes the risk of human life costs and socioeconomic costs, in a context of uncertainty about the end of the pandemic and that responds and solve two major problems: i) Targeting COVID-19 cases, allocating human and economic resources, and providing health and technical-medical treatment to reduce R_e_ and; ii) a plan of measures for the reactivation and revitalization of the economy, attending to this double curve [1] with multi-group variants that implies trade-offs [36] in the short, medium and long-term and in an intelligent way [37, 39].

### 4.5 The temperature has a negative impact on economic activity, but it is not statistically significant at 5%. However, the Granger test confirms that temperature causes economic activity

This statement is consistent with the literature review [29–31]. Also, there is recent evidence that seems to suggest that climatic conditions may influence the transmission of SARS-CoV-2 [80–82], however, there is no robust scientific evidence to conclusively affirm that the virus survives with heat and that The pandemic could lessen with the arrival of higher temperatures [83] and indirectly impact economic activity.

### 4.6 The macroeconomic model of aggregate supply and aggregate demand allows us to describe and explain that the negative impact of the systemic shocks of the COVID-19 on the level of economic activity is reinforced on both sides

Expansive monetary or fiscal policy measures will be successful if you have a long-term vision and attack the serious problem of the pandemic, and consider the structural characteristics, which is evidenced in the weak institutional framework (weak governance, expansion of informality, a dual economy with sectors with large differences in productivity, wages and salaries, unequal distribution of income, low levels of human capital, little investment in education and health), which promote precarious and low-quality services [34, 37].

For the new Keynesian School, the Peruvian case would be clear evidence where the State has had to intervene not only due to a precautionary principle as dictated by the Political Constitution but to face the Pandemic considered as a negative externality that affects the population. Faced with the market failure, the State seeks to solve short-term emergency problems to move in the direction of sustained growth for the long-term, based on human development [4, 13, 15, 23, 25, 41, 56, 57].

There is macroeconomic stability with an economy without inflation, however, it is appropriate to take advantage of the expansionary fiscal policy in the short, medium, and long-term to boost the economy. Attacking unemployment and fundamentally underemployment and motivating greater participation of the EAP (economically active population). The key business sector in Peru is 95% small and micro enterprises, which include approximately 70% informal. Expansive fiscal, monetary, and financial policies should be directed there, emphasizing in a policy of economic reactivation and expansion representative of most of the economic agents. Facilitate business and financial development services, through training programs and business capacity building, and temporary employment; and it will improve through COFIDE with credit expansion at preferential interest rates or close to zero for informal and vulnerable sectors. Reform and rescue bankrupt companies and institutions. Also, the financing of investments in access to fiber optics and the Internet to improve connectivity in sectors associated with TICs, services, reduce the digital divide, and, therefore, greater equality of opportunities that tend to reduce inequality of entry. And deal with structural reforms for the medium and long-term:

#### Governance

Given that there is an evident corruption that weighs down economic activity, and that requires political reforms and the judiciary, with proven cases of corruption and some associated with power groups, which press for policy measures that are not due to an economy of the free market but to a neo-mercantilist or rentier model that benefits only a few businessmen or a part of the country. The State of Emergency decreed by the Government and operated by the BCRP, skews the transfer policy and credit programs to the country's medium and large business sectors, excluding micro and small businesses that represent more than 95% of the business universe, including the informal sector of the country that was the most affected by the suppression policies. The pandemic has cost more than 20% of GDP, if you add the opportunity cost and citizen insecurity, the total economic cost would be catastrophic.

#### Duality and informality

Peru has a high informality (70% of the EAP). And the economic recovery has been more supportive of large companies. Peru is an economy that must serve the informal as a small businessman or entrepreneur, which would have a greater effect on economic reactivation. Fiscal, monetary, and financial policies should be directed and include the informal sector fundamentally. The Government Program "Reactiva Peru" which aims to avoid breaking the chain of payments to suppliers and massive layoffs, has been focused on groups that have a dominant position. According to the Government, as of May 29, 2020, only 3% of the Funds have been used for Micro Enterprises and 76% for medium and large enterprises.

Trusting that the profits of the economic activity of the big company *trickle-down* or revert, pull or drag the so-called reserve army of labor, of low productivity and income, condemns and weighs down our possibilities of achieving sustained economic growth. It is the deliberate support with expansive fiscal and monetary policies for the short term, as well as with long-term policies of investment in human capital and innovation to the informal, non-modern sector, with low levels of productivity and income, with important participation of the population in generating employment, which would generate sustained economic growth in the country. It is necessary to define policies for innovation and technological development in the short, medium, and long-term, prioritizing this sector, strengthening business capacities and with a monetary policy of greater inclusion and access to credit with preferential interest rates and fiscal policies for public investments that diversify the economy and expand the domestic market.

#### Precarious health and education services

There is a low stock of human capital in health and education (teachers, doctors, nurses, and technicians) and a stock of physical capital (infrastructure) that implies poor quality of public health and education service. There are 20% of the population living in poverty, 20% without access to drinking water from the public network, 35% without sanitary services, and 12% in overcrowding, which has increased due to the Pandemic. Public-private investment and regulatory entities that fulfill the function of making the economy more competitive can correct inequalities and generate quality public services with equal opportunities.

It is necessary to promote an Observatory for national and regional multidisciplinary entrepreneurship with the investigation of economic activity, related to sustainable human development, which implies the improvement of the quality of life, social welfare, and access to information in real-time, for the preparation of public policies with expert groups and decision-making. The observatory is also essential to address future risks of disasters or Pandemics.

## 5. Conclusions

The estimated econometric models have made it possible to present empirical evidence that supports and explains the dynamics and determinants of short-term economic activity, in a COVID-19 scenario in Peru. They are of practical use for the analysis of future trends; as well as for use for health prevention and the timely design of public, monetary, and fiscal policies, which, also, can prevent, mitigate and respond on time to the Pandemic, as well as affect the level of activity or economic recession. Policies with a vision of the future and must be consistent with short, medium, and long-term planning.

The public health policy implemented through molecular and serological diagnostic tests has been supported by the different governments of the world, as well as the implementation of better targeting and resource allocation strategies, which has led to mitigating and controlling the disease. The suppression strategy, although discriminated, has been important in this first stage because it has helped reduce the growth rate of the spread of COVID-19. However, in a second moment, a focused, effective and intelligent mitigation strategy must be implemented, which minimizes the risk of human life costs and socioeconomic costs, in a context of uncertainty about the end of the pandemic, and accompanied by economic policies that mitigate the economic recession, exacerbated by the underlying structural characteristics of the Peruvian economy: structural duality, weak institutions and governance, informality, underemployment, low-productivity, and low-incomes.

The Peruvian economy has the market, state, and civil society failures. There are privileged power groups that have resources and do not allocate scarce resources efficiently. It is necessary to promote the participation of civil society for the strengthening of social and institutional capital, with the fulfillment of norms and measures of social isolation. In the short term, reduce the number of infected, deaths and reactivate the economy; in the long term, promote sustained and inclusive economic growth based on investment and accumulation of human capital, and social and human development policies to achieve a better quality of life with equal opportunities for all Peruvians.

## Supporting information

S1 Model Theoretical, mathematical and econometric model. (PDF)

S1 Dataset Data panel ARDL model. Daily time series index, March to June 2020.
(XLSX)

S1 Annex Unit root of time series (level and first-difference). (DOCX)

S2 Annex Pairwise Granger causality tests (level). (DOCX)

## References

[1] Baldwin R. and Weder B., Mitigating the COVID Economic Crisis: Act Fast and Do Whatever It Takes, ed. Baldwin R. and Weder B. 2020: VoxEU.org CEPR Press 227.

[2] BCRP, COVID-19 y medidas del BCRP frente al la Pandemia. *En Revista Moneda* 182 2020: Lima, Peru. p. 86.

[3] Barro R.J. and King R.G., Time-separable preferences and intertemporal-substitution models of business cycles. The Quarterly Journal of Economics, 1984 99(4): p. 817–839.

[4] Eichenbaum, M.S., S. Rebelo, and M. Trabandt, Epidemics in the Neoclassical and New Keynesian Models. NBER Working Paper N° 27430, 2020. National Bureau of Economic Research(Jun 2020).

[5] Eichenbaum, M., S. Rebelo, and M. Trabandt, The Macroeconomics Epidemics. NBER Working Paper No 26882, 2020. National Bureau of Economic Research(March 2020).

[6] Eichenbaum, M.S., S. Rebelo, and M. Trabandt, *The Macroeconomics of Testing and Quarantining*. NBER Working Paper No 27104, 2020. National Bureau of Economic Research(May 2020).

[7] IMF, World Economic Outlook Update, June 2020. 2020. p. 20.

[8] IMF, World Economic Outlook: A Long and Difficult Ascent, October 2020. 2020. p. 204.

[9] Ferguson N., et al, Report 9: Impact of non-pharmaceutical interventions (NPIs) to reduce COVID-19 mortality and healthcare demand 2020, Faculty of Medicine. School of Public Health. Imperial College London COVID-19: Imperial College London p. 1–20.

[10] BCRP, Reporte de inflación: Panorama actual y proyecciones macroeconómicas 2020–2021. 2020: Lima. p. 152.

[11] Narayan, P.K., Reformulating Critical Values for the Bounds F-statistics Approach to Cointegration: An Application to the Tourism Demand Model for Fiji, M. University, Editor. 2004: Australia. p. 800.

[12] Pesaran M.H., Shin Y., and Smith R.J., Bounds testing approaches to the analysis of level relationships. Journal of Applied Econometrics, 2001 16(3): p. 289–326.

[13] Galí J., The State of New Keynesian Economics: A Partial Assessment. Journal of Economic Perspectives, 2018 32(3): p. 87–112.

[14] Woodford M., Interest and prices: Foundations of a theory of monetary policy 2003, USA: princeton university press 597.

[15] Mankiw N.G., Phelps E.S., and Romer P.M., The Growth of Nations. Brookings Papers on Economic Activity, 1995. 1995(1): p. 275–326.

[16] Barro R.J., Government Spending in a Simple Model of Endogeneous Growth. Journal of Political Economy, 1990 98(5): p. S103–S125.

[17] Domar E.D., Capital Expansion, Rate of Growth, and Employment. Econometrica, 1946 14(2): p. 137–147.

[18] Harrod R.F., An Essay in Dynamic Theory. The Economic Journal, 1939 49(193): p. 14–33.

[19] Kaldor N., A Model of Economic Growth. The Economic Journal, 1957 67(268): p. 591–624.

[20] Lucas R.E. Jr., On the mechanics of economic development. Journal of monetary economics, 1988 22(1): p. 3–42.

[21] Rebelo S., Long-Run Policy Analysis and Long-Run Growth. Journal of political Economy, 1991 99(3): p. 500–521.

[22] Romer P.M., Increasing Returns and Long-Run Growth. Journal of Political Economy, 1986 94(5): p. 1002–1037.

[23] Romer P.M., Endogenous Technological Change. Journal of Political Economy, 1990 98(5): p. S71–S102.

[24] Solow R.M., A Contribution to the Theory of Economic Growth. The Quarterly Journal of Economics, 1956 70(1): p. 65–94.

[25] Solow R.M., Technical change and the aggregate production function. The review of Economics and Statistics, 1957: p. 312–320.

[26] Barro, R.J., J.F. Ursúa, and J. Weng, The coronavirus and the great influenza pandemic: Lessons from the “spanish flu” for the coronavirus’s potential effects on mortality and economic activity. NBER Working Paper 26866, 2020.

[27] Barrios S., Bertinelli L., and Strobl E., Trends in rainfall and economic growth in Africa: A neglected cause of the African growth tragedy. The Review of Economics and Statistics, 2010 92(2): p. 350–366.

[28] Sangkhaphan S. and Shu Y., The Effect of Rainfall on Economic Growth in Thailand: A Blessing for Poor Provinces. Economies, 2020 8(1): p. 1.

[29] Dell M., Jones B.F., and Olken B.A., Temperature Shocks and Economic Growth: Evidence from the Last Half Century. American Economic Journal: Macroeconomics, 2012 4(3): p. 66–95.

[30] Kahn, M.E., et al., Long-term macroeconomic effects of climate change: A cross-country analysis. NBER Working Paper 26167, 2019.

[31] Masters, W.A. and M.S. McMillan., Climate and Scale in Economic Growth. CID Working Paper Series 2000.48, 2000(June).

[32] Bloom D.E., et al, Geography, Demography, and Economic Growth in Africa. Brookings Papers on Economic Activity, 1998 1998(2): p. 207–295. 12295931

[33] Everett, T., et al., Economic growth and the environment. MPRA Paper No. 23585, 2010(March 2010): p. 52.

[34] Gonzales J.R., et al, COVID-19 Pandemic and Public Health Policies in Peru: March-May 2020. Revista de Salud Pública, 2020 22(2)(April 30, 2020): p. 1–9. 10.15446/rsap.V22n2.87373 36753105

[35] Alvarez, F.E., D. Argente, and F. Lippi, A simple planning problem for COVID-19 lockdown. NBER Working Paper No 26981, 2020. National Bureau of Economic Research(April 2020).

[36] Acemoglu, D., et al., Optimal Targeted Lockdowns in a Multi-Group SIR Model. NBER Working Paper No 27102, 2020. National Bureau of Economic Research(May 2020).

[37] Loayza N.V., Costs and Trade-Offs in the Fight Against the COVID-19 Pandemic: A Developing Country Perspective, R.P.B.F.t.W.B.M. Hub, Editor. 2020, World Bank: USA.

[38] Spence M. and Long C., *Graphing the Pandemic Economy*, in *On Point* 2020: Project Syndicate.

[39] Loayza N. and Pennings S., Macroeconomic Policy in the Time of COVID-19 2020, World Bank.

[40] MEF MEF publica lista de las 71,553 empresas que accedieron al programa “Reactiva Perú” hasta fines de mayo 2020, Ministerio de Economía y Finanzas (MEF): Lima, Peru.

[41] Galí J., Monetary policy, inflation, and the business cycle: an introduction to the new Keynesian framework and its applications 2015: Princeton University Press.

[42] Jones, C.J., T. Philippon, and V. Venkateswaran, Optimal mitigation policies in a pandemic: Social distancing and working from home. NBER Working Paper N° 26984, 2020. National Bureau of Economic Research(April 2020).

[43] Diewert, W.E. and K.J. Fox, Measuring Real Consumption and CPI Bias under Lockdown Conditions. NBER Working Paper No. 27144, 2020. National Bureau of Economic Research(May 2020).

[44] Coibion, O., Y. Gorodnichenko, and M. Weber, The Cost of the Covid-19 Crisis: Lockdowns, Macroeconomic Expectations, and Consumer Spending. NBER Working Paper No. 27141, 2020. National Bureau of Economic Research(May 2020).

[45] Narita F. and Yin R., In Search of Information: Use of Google Trends' Data to Narrow Information Gaps for Low-income Developing Countries 2018, International Monetary Fund.

[46] Götz T.B. and Knetsch T.A., Google data in bridge equation models for German GDP. International Journal of Forecasting, 2019 35(1): p. 45–66.

[47] Eichenauer, V., et al., Constructing Daily Economic Sentiment Indices Based on Google Trends. KOF Working Papers, 2020. 484.

[48] Shin S.-Y., et al, High correlation of Middle East respiratory syndrome spread with Google search and Twitter trends in Korea. Scientific Reports, 2016 6(1): p. 32920 10.1038/srep32920 27595921 PMC5011762

[49] Gluskin R.T., et al, Evaluation of Internet-Based Dengue Query Data: Google Dengue Trends. PLOS Neglected Tropical Diseases, 2014 8(2): p. e2713 10.1371/journal.pntd.0002713 24587465 PMC3937307

[50] Chiu A.P.Y., Lin Q., and He D., News trends and web search query of HIV/AIDS in Hong Kong. PLOS ONE, 2017 12(9): p. e0185004 10.1371/journal.pone.0185004 28922376 PMC5602633

[51] Greenwood J., et al, An equilibrium model of the African HIV/AIDS epidemic. Econometrica, 2019 87(4): p. 1081–1113.

[52] *Decreto De Urgencia N° 090**–*2020 Decreto De Urgencia Que Establece Medidas Excepcionales Y Temporales Que Coadyuven Al Cierre De Brechas De Recursos Humanos En Salud Para Afrontar La Pandemia Por La Covid -19, N. Legales, Editor. 2020, Diario Oficial El Peruano: Lima, Perú.

[53] *Decreto Legislativo N° 1276 Que Aprueba El Marco De La Responsabilidad Y Transparencia Fiscal Del Sector Público No Financiero*, N. Legales, Editor. 2020, Diario Oficial El Peruano: Lima, Perú.

[54] Seminario B., Sanborn C., and Alva N., *Cuando despertemos en el 2062: visiones del Perú en 50 años*. 2012: Universidad del Pacífico.

[55] Seminario B., *Esta pandemia cierra el periodo neoliberal*, in Ojo Público 2020: Lima, Perú.

[56] Samuelson P.A., An Exact Consumption-Loan Model of Interest with or without the Social Contrivance of Money. Journal of Political Economy, 1958 66(6): p. 467–482.

[57] Stiglitz J.E. and Rosengard J.K., *Economics of the public sector: Fourth international student edition*. 2015: WW Norton & Company 10.1177/1071100714555713

[58] Gonzales, J.R. and L. Varona, Economic growth and the impact on poverty and inequality in income distribution: Peru 1985–2017., in Working paper CrecedPeru, prepared for X RIDGE Forum—Workshop on Poverty and inequality and Pacifico University., CrecedPeru, Editor.: Lima.

[59] Varona L. and Gonzales J.R., Crecimiento Económico y Distribución del Ingreso: Perú 1985–2017 Problemas Del Desarrollo. Revista Latinoamericana De Economía, 2020 69636.

[60] IDB, Public Policy to Tackle Covid-19 Recommendations for Latin America and the Caribbean 2020.

[61] CEPAL, Dimensionar los efectos del COVID-19 para pensar en la reactivación, in *Informe Especial N° 2 COVID-19*. 2020, CEPAL: Santiago, Chile, p. 21.

[62] CEPAL, Estudio Económico de América Latina y el Caribe Principales condicionantes de las políticas fiscal y monetaria en la era pospandemia de COVID-19, in COVID-10. Respuesta 2020: Santiago, Chile p. 213.

[63] Pesaran, M.H. and Y. Shin, An autoregressive distributed-lag modelling approach to cointegration analysis, in Econometric Society Monographs, Cambridge, Editor. 1997. p. 371–413.

[64] Pesaran, B. and M. Pesaran. Time series econometrics using Microfit 5.0. 2009.

[65] Narayan P.K., The saving and investment nexus for China: evidence from cointegration tests. Applied economics, 2005 37(17): p. 1979–1990.

[66] Arrow K.J., The Economic Implications of Learning by Doing. The Review of Economic Studies, 1962 29(3): p. 155–173.

[67] Costello D.M., A cross-country, cross-industry comparison of productivity growth. Journal of Political Economy, 1993 101(2): p. 207–222.

[68] Céspedes N., Lavado P., and Ramírez N., Productividad en el Perú: Medición, determinantes e implicancias (Capítulo 2), ed. U.d. Pacifico 2016, Lima, Perú.

[69] Ocaña Victor, R., J.R. Gonzales, and L. Varona, Forecast of the prevalence of the cases of the Dengue virus with the application of models ARIMA in Piura-Peru 2013–2016., in Working paper CrecedPeru, CrecedPeru, Editor. 2018: Peru.

[70] Hethcote H.W., The mathematics of infectious diseases. SIAM review, 2000 42(4): p. 599–653.

[71] Kermack W.O. and McKendrick A.G., A contribution to the mathematical theory of epidemics. Proceedings of the royal society of london, 1927 115(772): p. 700–721.

[72] Casella F., Can the COVID-19 epidemic be managed on the basis of daily data? arXiv, 2020: p. arXiv: 2003.06967.

[73] Walker P.G.T., et al, The impact of COVID-19 and strategies for mitigation and suppression in low- and middle-income countries. Science, 2020 369(6502): p. 413–422. 10.1126/science.abc0035 32532802 PMC7292504

[74] Atkeson, A., What Will Be the Economic Impact of COVID-19 in the US? Rough Estimates of Disease Scenarios. NBER Working Paper No. 26867, 2020. National Bureau of Economic Research(March 2020).

[75] BCRP, El Banco Central de Reserva del Perú realizó la segunda subasta de Repos con. Carteras Garantizadas 2020, Banco Central de Reservas del Peru: Lima, Perú p. 1.

[76] Caballero, R.J. and A. Simsek, Asset prices and aggregate demand in a "Covid-19" shock: A model of endogenous risk intolerance and LSAPS. NBER Working Paper 27044 2020. National Bureau of Economic Research(April 2020).

[77] Thirlwall A.P., The balance of payments constraint as an explanation of international growth rate differences. PSL Quarterly Review, 2011 64(259): p. 429–438.

[78] BCRP, Resumen Informativo Semanal 2020, Banco Central de Reservas del Peru (BCRP) Lima, Peru p. 16.

[79] Topcu M. and Gulal O.S., The impact of COVID-19 on emerging stock markets Finance Research Letters, 2020: p. 101691. 10.1016/j.frl.2020.101691 PMC7348595 32837378

[80] Menebo M.M., Temperature and precipitation associate with Covid-19 new daily cases: A correlation study between weather and Covid-19 pandemic in Oslo, Norway Science of The Total Environment, 2020: p. 139659. 10.1016/j.scitotenv.2020.139659 PMC7258804 32492607

[81] Wang J., et al, High temperature and high humidity reduce the transmission of COVID-19. SSRN 3551767, 2020.

[82] Sajadi M.M., et al, Temperature and latitude analysis to predict potential spread and seasonality for COVID-19. SSRN 3550308, 2020 10.2139/ssrn.3550308 32525550 PMC7290414

[83] Bukhari Q. and Jameel Y., Will coronavirus pandemic diminish by summer? SSRN 3556998, 2020.

[84] Yang P., et al, Feasibility study of mitigation and suppression strategies for controlling COVID-19 outbreaks in London and Wuhan. PLOS ONE, 2020 15(8): p. e0236857 10.1371/journal.pone.0236857 32760081 PMC7410247

[85] Pesaran, M.H. and Y. Shin, An autoregressive distributed-lag modelling approach to cointegration analysis, in Econometrics and Economic Theory in the 20‴ Century: the Ragnar Frisch Centennial Symposium., A.C. Steinar Strøm, Matthew Jackson, Editor. 1998: Cambridge University. p. 371–413.

[86] Wooldrige, J., Introducción a la econometría: Un enfoque moderno, ed. T. Learning. 2003.

[87] Gujarati D. and Porter D., Econometría (5ta ed) 2010, California, Estados Unidos: Mc Graw Hill.

