# Supplementary material for: Dynamics of the impact of COVID-19 on the economic activity of Peru
Source: PLoS One. 2021 Jan 8;16(1):e0244920. doi: 10.1371/journal.pone.0244920 (PMC7793288; doi:10.1371/journal.pone.0244920)
Supplement: S1 Model — (PDF) [file pone.0244920.s001.pdf]

Theoretical, mathematical and econometric model

Theoretical and mathematical model

The assumptions

In relation to the subsystem of the population facing a COVID-19 pandemic, the following assumptions are assumed following the methodology [1, 2] and adapted [3].

- The total population N is constant and is divided into 3 categories. i) the susceptible individuals (S_t) at the initial moment are the healthy population that can become infected. ii) infectious individuals (I_t) can infect susceptible individuals. iii) recovered individuals (R_t), acquire immunity, and no longer have the transmission capacity.
- The birth and death rates do not vary. Immigration and emigration are not considered.
- At the start of the epidemic outbreak, only a small percentage of the population is infected $I_o = 1/N$
- At the initial moment, the number of susceptible and infected is positive.
- The interaction between S, I, and R individuals is that susceptible individuals become infectious and these become recovered or dead individuals.

About the economic subsystem, the following assumptions are made based on the aggregate supply and aggregate demand model [4]:

- A short-term model, in which prices are rigid. The prices of goods and services are fixed.
- The components of aggregate demand (DA) are given by the consumption of goods and services (C), investments and services (I), public spending (G) and net exports of goods and services (XN). Then the aggregate demand remains: DA = C + I + G + XN.

  Aggregate Demand is assumed to be affected by an exogenous disturbance λ. We consider the COVID-19 pandemic to be the exogenous shock or the shock that exogenously affects aggregate demand. Thus: DA = Y = DA (C, I, XN) + G + λ. Consumption level C depends on price level p, real income level y, real interest rate i, real wage w, income tax rate t. It remains: C = C (p, y, i, w, t). Investment level I depends

on the price level p, on the real interest rate i, on the real price of the shares pa, on the real exchange rate e. It remains: I = I (p, y, i, pa, e). The level of public spending G is exogenous and determined by the Government. It remains: G = g. Net exports XN, depends on the price level p, real exchange rate e, real interest rate r, price of minerals and real energy pm *, real wage w and world income y *. It remains: XN = XN (p, e, i, w, y *, pm *).

- The Aggregate Supply (SA) depends on the price level p, real wage w, real interest rate r, real exchange rate e, climatic conditions c and technology τ. The COVID-19 pandemic is also considered to be an exogenous shock or the shock that exogenously affects aggregate supply. Thus Y = SA (p, w, i, e, c, τ) + λ

The structural form

The subsystem of the population facing a pandemic is reduced to 4 equations. Where, equation (1) represents the dynamics of individuals infected by the Pandemic.

$$\dot{I} = -\frac{\beta IS}{N} \quad \text{... (1)}$$

In equation (2) represents the change in the susceptible population:

$$\dot{S} = \frac{\beta\, SI}{N} - \gamma I \quad \text{... (2)}$$

Equation (3) represents the change in the recovered population:

$$\dot{R} = \gamma\, I \quad \text{... (3)}$$

The economic subsystem can be expressed in 6 equations that represent the aggregate supply and aggregate demand of the Peruvian economy:

$$y = DA = DA(C, I, XN) + G + \lambda \quad \text{... (4)}$$

$$C = C(p, y, i, w, e, t) \quad \text{... (5)}$$

$Cp < 0$, $0 < Cy < 1$, $Ci < 0$, $Cw > 0$, $Ce < 0$, $Ct < 0$

$$I = I(p, y, i, e, pa) \quad \text{... (6)}$$

$Iy > 0$, $Ip < 0$, $Ii < 0$, $Ie < 0$, $Ipa > 0$

$$G = \bar{g} \quad \text{... (7)}$$

$$XN = XN(p, w, i, e, y *, pm *) \quad ...(8)$$

$XNp < 0$, $XNi < 0$, $XNw < 0$, $XNe > 0$, $XNy *> 0$, $XNpm *> 0$

$$y = SA = SA(p, w, i, e, c, \tau) + \lambda \quad ...(9)$$

$$N = S + I + R \quad ...(10)$$

The reduced form

Equation (1) represents the dynamics of the infected that increases to the infection rate (β), as the number of susceptible individuals that have been infected increases and decreases to the recovery rate (γ) as the infected recover or die.

γ= It is the recovery rate, is the speed with which an individual goes from infected to recovered or the rate at which infected individuals recover or die. This rate represents the inverse of the average number of days that COVID-19 lasts, which is considered by experts to be 14 days.

From equation (10), R is cleared and the population subsystem facing the Pandemic can be reduced to a system of two equations, equation (1) and (2).

Assuming that S is constant and approximately equal to N under initial conditions. This assumption decouples equation (2), from equation (1), S = N and equation (2) remains as:

$$\dot{I} = \frac{\beta\, IN}{N} - \gamma I$$

Given: N = S, replacing

$\dot{I} = \beta I - \gamma I,$ is factored:

$$\dot{I} = (\beta - \gamma)I \quad ...(11)$$

The growth rate of the infection will be:

$$\frac{\dot{I}}{I} = (\beta - \gamma) \quad ...(12)$$

From the differential equation (11), the solution or path for the infected population is derived:

$$I_t = I_o e^{(\beta-\gamma)t} = I_o e^{rt} \quad ...(13.\text{a})$$

Where, r=β-ϒ. It is shown that the doubling time of infectious T_d cases is given by:

$$T_d = \frac{ln2}{\beta - \gamma} = \frac{ln2}{r} \quad ...(13.\text{b})$$

The epidemic ends as S_t falls below $\rho = \frac{\gamma}{\beta}$, which is derived from the epidemic balance $\dot{I} = 0$ ($\gamma > \beta$). In summary, the epidemic ends because of the lack of newly infected individuals and not because of the lack of susceptible individuals.

Theorem: If $R_e \leq 1$, then It monotonically decreases to zero as $t \Rightarrow \infty$. From this it is inferred: If $R_e > 1$, the level of infected individuals It begins to increase, reaches its maximum, and then decreases to zero as $t \Rightarrow \infty$. This scenario of increasing numbers of infected individuals is called an epidemic. It follows that an infection can cause an epidemic in a susceptible population if $R_e > 1$ or $\beta > \gamma$ or $\beta > \kappa * \tau$. Then the effective number R_e is obtained as:

$$R_e = \frac{\beta}{\gamma} = \frac{\kappa * \tau}{\gamma} = \lambda \qquad \ldots (14)$$

$\kappa =$ number of contacts of each individual per unit of time (day).

$\tau =$ transmissibility of infectious disease. It is a fraction of contacts that result in the transmission of the disease.

$R_{e=}$ reflects the population of susceptible people who are infected and, therefore, would stop working, producing and receiving income, affecting supply and aggregate demand. Thus, it is considered that $\lambda = R_e$ is a parameter or variable of exogenous disturbance and, according to its magnitude, generates supply and aggregate demand shocks, by affecting employment and the level of production and income of the economy.

If $\lambda = R_e > 1$, it implies that the pandemic continues to generate and increase the number of infected and dead individuals, affecting supply and aggregate demand. If $\lambda = R_e < 1$, it implies that the pandemic is being controlled and the economy can begin to reactivate by increasing employment, production and income by affecting the aggregate demand and supply of the economy. If an epidemiological balance is assumed, such that $I^{\cdot} = 0$, this implies that there is no longer an infected population. Equation (2) is expressed as:

$$\beta \frac{SI}{N} - \gamma I \ = 0$$

$$\frac{S}{N} = \frac{\gamma}{\beta} = \frac{1}{\lambda} \qquad \ldots (15)$$

This would be the inverse of the effective reproductive number. This implies that for a $\lambda = R_e$>1, the number of infected people increases, and simultaneously the percentage of the susceptible population with respect to the total population is reduced, because it is being infected, leading to a Pandemic. And if $\lambda = R_e$<1, this implies that the percentage of the susceptible, healthy, or immune population is increasing and simultaneously the number of infected people is decreasing. This would affect the population that would be willing to work, increasing employment, production, and income in the economy, which would affect supply and aggregate demand negatively.

In the economic subsystem, replacing equation (2), (3) and (5) in equation (1), it remains:

$$y = DA = DA\big(C(p, y, i, w, e, t), I(p, y, i, e, pa), XN(p, i, w, e, y*, pm*)\big) + g + \lambda \quad ...(16)$$

replacing equation (14), we obtain the aggregate demand function:

$$y = DA(p, y, i, w, e, t, pa, y*, pm*) + g + Re \quad ...(17)$$

Where: $DAp > 0$, $DAy > 0$, $DAi < 0$, $DAw > 0$, $DAe >< 0$, $DAt < 0$, $DApa > 0$, $DAy *> 0$, $DAg > 0$, $DARe < 0$, $DApm* > 0$

The aggregate supply function according to equation (6), is expressed as:

$$y = SA = SA(p, w, i, e, c, \tau) + Re \qquad ...(18)$$

Where: $SAp > 0$, $SAw < 0$, $SAi < 0$, $SAe > 0$, $SAc < 0$, $SARe < 0$, $SA\tau > 0$

Given equations (17) and (18) we derive the reduced form of the model:

$$y = y(i, w, e, t, pa, y*, pm*, c, \tau, g, Re) \qquad ...(19)$$

and

$$p = p(i, w, e, t, pa, y*, c, \tau, g, Re) \qquad ...(20)$$

Then, the function of the determinants of the level of economic activity could be estimated econometrically in equation (19). However, the availability of information suggests that we dispense with some operational variables such as real wages w, income tax t, level of international economic activity y*, and technology τ. For the price of the pa shares, the operational variable General Stock Index provided by the BCRP called igb is used. For climate c, the Lima temperature operational variable provided by SENANHI called tem is used. For Government expense g we

use the total number of samples provided by MINSA called prut. For international prices pm * we use the international price of the main mining export product that is copper as the operational variable, given by the BRCP called cob. We add a dummy variable associated with the application of the suppression strategy and subsequent gradual opening of economic activities implemented by the Government d1. Then, the causal relationships of economic activity in the short term would be:

$$\frac{\partial y}{\partial i} < 0, \frac{\partial y}{\partial e} <> 0, \frac{\partial y}{\partial igb} > 0, \frac{\partial y}{\partial tem} < 0, \frac{\partial y}{\partial prut} > 0, \frac{\partial y}{\partial Re} < 0, \frac{\partial y}{\partial cob} > 0, \frac{\partial y}{\partial d1} < 0$$

Equation (20) is specified in logarithms, it is then:

$$lny = \beta_0 + \beta_1 ln(i) + \beta_2 ln(e) + \beta_3 ln(igb) + \beta_4 ln(tem) + \beta_5 ln(prut) + \beta_6 ln(Re) + \beta_7 ln(cob) + \beta_8 ln(d1) + \varepsilon \quad \ldots (20.a)$$

Econometric model

The Autoregressive Distributed Lags (ARDL) model analyzes time series, where the dependent and independent variables are related simultaneously and by lagged values [5-7]. The general ARDL model (p, q_1, q_2, q_3 ... q_k), is given by y_t which is an endogenous variable, and x_1, x_2, x_3... x_k, are the exogenous variables, expressed in equation (21).

$$y_t = \alpha_0 + \alpha_1 t + \sum_{i=1}^{p} \psi_i y_{t-i} + \sum_{j=1}^{k} * \sum_{l_j=0}^{q_j} \beta_{j,l_j} x_{j,t-lj} + \varepsilon_t \quad \ldots (21)$$

Where: ε_t are innovations, α_0 is a constant term, α_1 is the coefficient associated with a linear trend, ψ_i is the coefficient associated with lags of y_t, y $\beta_{j,lj}$ are the coefficients associated with lags of k regressors $x_{t,j}$ for $j= 1,2,3,...,k$. It can be written (22).

$$\psi_i(L) y_t = \alpha_0 + \alpha_1 t + \sum_{j=1}^{k} \beta_j (L) x_{j,t} + \varepsilon_t \quad \ldots (22)$$

Where: L denotes the lag operator and defines $\psi_i(L)$ y $\beta_j(l)$ as the polynomial lag. The starting point for ARDL applications is the estimation of intertemporal dynamics. According to the methodology [5] the relationship between $\mathbf{y_t}$ is estimated both in its lags and in the contemporary

and lagged values of the k regressors $x_{j,t}$, proposed in the ARDL model. Equation (22) is converted to the intertemporal dynamic regression, equation (23).

$$y_t = \alpha_0 + \alpha_1 t + \sum_{i=1}^{p} b_{0,i}\, y_{t-i} + \sum_{j=1}^{k} b_j\, x_{j-t} + \sum_{j=1}^{k} * \sum_{l_j=1}^{q_j-1} c_{j,}\, l_j \Delta x_{j,t-l} + \varepsilon_t \; \ldots (23)$$

ARDL models are estimated by ordinary least squares (OLS). The optimal model is estimated according to the Schwarz criterion. The short-term analysis finds a long-term Cointegration relationship between the exogenous variables and the endogenous variable. It is tested whether there is a long-term relationship.

Then, the limit test is important to determine the cointegration ratio of the error correction model (CE). An F test is used with the Ho hypothesis, that the variables are not cointegrated. The test statistic is compared with two critical values for cases where the variables are purely I (0) or I (1). When the test statistic is above the upper critical value, the null hypothesis (Ho) is rejected and concludes that cointegration is possible and a CE model can be estimated. The series do not have a unit root, that is, they are stationary and it is achieved with the first difference, that is, they are integrated of order I (1).

Also, after the elaboration of the stylized facts, the Granger test of causality is considered [8]. That there is a correlation between two variables does not imply causality, that is, that one variable is correlated with another does not always imply that one of them is the cause of the changes in the values of another. Granger was the first to propose a causality test, because the past can affect the future. Specifically, the test seeks to know if there is evidence to affirm that there is a long-term causal relationship of COVID-19 on economic activity. The null hypothesis would be: H_o: that said causality does not exist or H_a: that we do not know if said causality does not exist.

Data

The variables are in the index, with a base period for March 6 equal to 100. Which is from the first confirmed case of COVID-19 in Peru. Two economic variables are used for economic activity. The first is based on the average daily energy demand in kilowatt hours, KW (see: www.coes.org.pe/Portal/portalinformacion/demanda). The second, based on mobility data

generated by Google, estimates a typical production function of capital and labor, doing the Google search for employment and investment (see: https://trends.google.com). Then a weighted index is calculated as a productive factor of 45% and 55% respectively.

As the COVID-19 operational variable, the basic propagation number (R_e) is used. The estimated data of the Re indicator, are calculated from the SIR model methodology following the available methodology and adapted for the Peruvian case [3]. With this methodology, the 94 data were completed, making a regression and calculating Re according to the estimated parameters. Therefore, estimates are made with a sample of 62 and 94, respectively, because the most recent sample is expected to have more significant results compared to the first month, which was more volatile and had more noise.

The operational variables General Stock Index of the Lima Stock Exchange, the data of the price at the end of the day is considered and the international price of copper is similar; the reference interest rate and the exchange rate are adjusted for inflation, the same as those provided by the Central Reserve Bank of Peru (BCRP). https://estadisticas.bcrp.gob.pe/estadisticas/series/. For the Lima temperature operational variable provided by SENANHI. https://senamhi.gob.pe/. For the total number of diagnostic tests (rapid and molecular tests) provided by the Ministry of Health (MINSA). https://www.datosabierto.gob.pe/search/field_topic/covid-19-917 or Wikipedia that is updated with MINSA data: https://es.wikipedia.org/wiki/Pandemia_de_enfermedad_por_coronavirus_de_2020_en_Per%C3%BA

In summary, it is expected to estimate 4 ARDL models.

References

1. Casella, F. *Can the COVID-19 epidemic be controlledon the basis of daily test reports?* Physics and Society Cornell University 2020; Available from: https://arxiv.org/abs/2003.06967.

2. Hethcote, H.W., *The Mathematics of Infectious Diseases.* SIAM Review, 2000. 42(4): p. 599-653.
3. Gonzales-Castillo, J.R., et al., *Pandemia de la COVID-19 y las Políticas de Salud Pública en el Perú: marzo-mayo 2020.* Revista de Salud Pública, 2020. 22(2): p. 1-9.
4. Dornbusch, R., S. Fischer, and R. Startz, *Macroeconomics*. McGraw-Hill Higher Education ed. 2011. 640.
5. Pesaran, M.H. and Y. Shin, *An autoregressive distributed lag modelling approach to cointegration analysis.* , C.C.U. Press, Editor. 1997: En S. Strom (Ed.).
6. Pesaran, H., Y. Shin, and R. Smith, *Bounds testing approaches to the analysis of level relationships.* Journal of Applied Econometrics, 2001. 16(3): p. 289-326.
7. Narayan, P.K., *Reformulating critical values for the bounds F-statistics approach to cointegration: An application to the tourism demand model for Fiji.*, in *Discussion Papers*, D.o.E. Monash University, Editor. 2004.
8. Granger, C.W.J., *Investigating Causal Relations by Econometric Models and Cross-spectral Method.* Econometrica, 1969. 37(3)(August 1969): p. 424-438.
