# Supplementary material for: Dynamics of the impact of COVID-19 on the economic activity of Peru
Source: PLoS One. 2021 Jan 8;16(1):e0244920. doi: 10.1371/journal.pone.0244920 (PMC7793288; doi:10.1371/journal.pone.0244920)
Supplement: S1 Annex — (DOCX) [file pone.0244920.s003.docx]

**S1 Annex:** **Unit root of time series** (level and first-difference)

| Methods ^1^ | Variable level | t-Statistic (Prob) | (C,T,L/B)^2^ | Variable 1st-difference  Variable | t-Statistic (Prob^1^.) | (C,T,L/B)^2^ |
| --- | --- | --- | --- | --- | --- | --- |
| ADF | **ln(Ye)** | -1.8062  (.6898) | (C,T,10) | **Δln(Ye)** | -1.9803^**^  (.0463) | (0,0,10) |
| PP |  | -1.9974  (.2873) | (C,0,3) |  | -11.3671^***^  (.0000) | (0,0,12) |
| KPSS |  | .2322  (—) | (C,T,4) |  | .2137^***^  (—) | (C,0,13) |
| ADF | **ln(Yi)** | -1.1862  (.9380) | (0,0,4) | **Δln(Yi)** | -8.7960^***^  (.0000) | (0,0,3) |
| PP |  | -1.2285  (.9426) | (0,0,3) |  | -26.1937^***^  (.0001) | (C,0,3) |
| KPSS |  | .8773  (—) | (C,T,3) |  | .3479^**^  (—) | (C,0,3) |
| ADF | **ln(Re)** | -1.0321  (.9316) | (C,T,0) | **Δln(Re)** | -6.4673^***^  (.0000) | (0, 0,0) |
| PP |  | -1.2074  (.9001) | (C,T,3) |  | -6.7173^***^  (.0000) | (0, 0,3) |
| KPSS |  | .1621  (—) | (C,T,6) |  | .1930^***^  (—) | (C,0,2) |
| ADF | **ln(pru)** | -.8366  (.8892) | (C,T,2) | **Δln(pru)** | -8.6017^***^  (.0000) | (0,0,1) |
| PP |  | -.7515  (.8740) | (C,T,7) |  | -11.7804^***^  (.0000) | (0,0,6) |
| KPSS |  | .1359  (—) | (C,T,2) |  | .2690^***^  (—) | (C,0,8) |
| ADF | **ln(i)** | -1.6241  (.0979) | (0,0,0) | **Δln(i)** | -7.8102^***^  (.0000) | (0,0,0) |
| PP |  | -1.6241  (.0979) | (0,0,0) |  | -7.8102^***^  (.0000) | (0,0,0) |
| KPSS |  | .3890  (—) | (C,0,2) |  | .3045^***^  (—) | (C,0,1) |
| ADF | **ln(e)** | -1.9939  (.5930) | (C,T,0) | **Δln(e)** | -7.8927^***^  (.0000) | (0,0,0) |
| PP |  | -2.0324  (.5722) | (C,T,1) |  | -7.9037^***^  (.0000) | (0,0,5) |
| KPSS |  | .1516  (—) | (C,T,6) |  | .0992^***^  (—) | (C,0,6) |
| ADF | **ln(cob)** | -2.1869  (.2130) | (C,0,0) | **Δln(cob)** | -8.5745^***^  (.0000) | (0,0,0) |
| PP |  | -2.1213  (.2372) | (C,0,3) |  | 8.8847^***^  (.0000) | (0,0,5) |
| KPSS |  | .8979  (—) | (C,0,6) |  | .0959^***^  (—) | (C,0,9) |
| ADF | **ln(igb)** | -3.0994  (.1155) | (C,T,0) | **Δln(igb)** | -8.4058^***^  (.0000) | (0,0,0) |
| PP |  | -3.0994  (.1155) | (C,T,0) |  | -8.7329^***^  (.0000) | (0,0,6) |
| KPSS |  | .9779  (—) | (C,0,5) |  | .0992^***^  (—) | (C,0,7) |
| ADF | **ln(tem)** | -1.2730  (.6368) | (C,0,1) | **Δln(tem)** | -11.6582^***^  (.0000) | (0,0,0) |
| PP |  | -1.4860  (.5341) | (C,0,2) |  | -12.0719^***^  (.0000) | (0,0,2) |
| KPSS |  | .9003  (—) | (C,0,6) |  | .0445^***^  (—) | (C,0,5) |

Source: Own Elaboration. 1/ The unit root test methods used are: Dickey-Fuller-Augmented (ADF), Phillips-Perron (PP), and Kwiatkowski-Phillips-Schmidt-Shin (KPSS) respectively. The null hypothesis of the ADF test and the PP test is that the series has a unit root, the null hypothesis of the KPSS test is that the tested series is stationary. 2 / (C, T, L / B) refers to the intercept, trend, and delay length (ADF) / bandwidth (Bartlett Kernel of PP) specified in the tests. Intercept and trend by experimentation in EViews. It automatically selects the delay length.
