# Supplementary material for: Dynamics of the impact of COVID-19 on the economic activity of Peru
Source: PLoS One. 2021 Jan 8;16(1):e0244920. doi: 10.1371/journal.pone.0244920 (PMC7793288; doi:10.1371/journal.pone.0244920)
Supplement: S2 Annex — (DOCX) [file pone.0244920.s004.docx]

**S2 Annex: Pairwise Granger Causality Tests** (level)

| **Null Hypothesis:** | | **F-Statistic**  **1/**  n=62 | **F-Statistic**  **2/**  n=93 | |
| --- | --- | --- | --- | --- |
| ln(R_e_) does not Granger Cause LOG(Ye) | | 8.7677***  [.0044] | 5.3015**  [.0236] | |
| ln(pru) does not Granger Cause LOG(Ye) | | .0065  [.9359] | .0113  [.9154] | |
| ln(i) does not Granger Cause LOG(Ye) | | .8066  [.3728] | .6315  [.4289] | |
| ln(e) does not Granger Cause LOG(Ye) | | 3.6193*  [.0620] | .2991  [.5858] | |
| ln(cob) does not Granger Cause LOG(Ye) | | 6.2937**  [.0149] | 4.2338**  [.0425] | |
| ln(igb) does not Granger Cause LOG(Ye) | | 5.4716**  [.0227] | 8.1774***  [.0053] | |
| ln(tem) does not Granger Cause LOG(Ye) | | 4.4755**  [.0386] | .4216  [.5178] | |
|  | |  |  | |
| ln(R_e_) does not Granger Cause LOG(Yi) | 17.9352***  [.0001] | 26.6167***  [.0000] |  |  |
| ln(pru) does not Granger Cause LOG(Yi) | 8.0174***  [.0063] | 1.8392  [.1784] |  |  |
| ln(i) does not Granger Cause LOG(Yi) | .5070  [.4792] | 2.2566  [.1365] |  |  |
| ln(e) does not Granger Cause LOG(Yi) | 15.2706***  [.0002] | 3.5925*  [.0612] |  |  |
| ln(cob) does not Granger Cause LOG(Yi) | 8.9998***  [.0040] | 19.3853***  [.0000] |  |  |
| ln(igb) does not Granger Cause LOG(Yi) | 13.8673***  [.0004] | 18.5206***  [.0001] |  |  |
| ln(tem) does not Granger Cause LOG(Yi) | 13.7736***  [.0005] | 7.3907***  [.0079] |  |  |

Source: Own elaboration. The Granger Causality method is applied with a lag for two data samples. n is the number of observations; F is the F-statistic, and p is the probability. 1/ n=62, lags: 1; sample: 07/04/2020 07/06/2020. 2/ n=93, lags: 1; sample: 06/03/2020 07/06/2020.
